# MGFM: a novel tool for detection of tissue and cell specific marker genes from microarray gene expression data

**DOI:** 10.1186/s12864-015-1785-9

**Published:** 2015-08-28

**Authors:** Khadija El Amrani, Harald Stachelscheid, Fritz Lekschas, Andreas Kurtz, Miguel A. Andrade-Navarro

**Affiliations:** Charité - Universitätsmedizin Berlin, Berlin Brandenburg Center for Regenerative Therapies (BCRT), Berlin, 13353 Germany; Berlin Institute of Health, Berlin, 10117 Germany; Seoul National University, College of Veterinary Medicine and Research Institute for Veterinary Science, Seoul, 151-742 Republic of Korea; Faculty of Biology, Johannes Gutenberg University of Mainz, Mainz, Germany; Institute of Molecular Biology, Mainz, Germany

**Keywords:** Microarrays, Marker genes, Samples

## Abstract

**Background:**

Identification of marker genes associated with a specific tissue/cell type is a fundamental challenge in genetic and cell research. Marker genes are of great importance for determining cell identity, and for understanding tissue specific gene function and the molecular mechanisms underlying complex diseases.

**Results:**

We have developed a new bioinformatics tool called MGFM (Marker Gene Finder in Microarray data) to predict marker genes from microarray gene expression data. Marker genes are identified through the grouping of samples of the same type with similar marker gene expression levels. We verified our approach using two microarray data sets from the NCBI’s Gene Expression Omnibus public repository encompassing samples for similar sets of five human tissues (brain, heart, kidney, liver, and lung). Comparison with another tool for tissue-specific gene identification and validation with literature-derived established tissue markers established functionality, accuracy and simplicity of our tool. Furthermore, top ranked marker genes were experimentally validated by reverse transcriptase-polymerase chain reaction (RT-PCR). The sets of predicted marker genes associated with the five selected tissues comprised well-known genes of particular importance in these tissues. The tool is freely available from the Bioconductor web site, and it is also provided as an online application integrated into the CellFinder platform (http://cellfinder.org/analysis/marker).

**Conclusions:**

MGFM is a useful tool to predict tissue/cell type marker genes using microarray gene expression data. The implementation of the tool as an R-package as well as an application within CellFinder facilitates its use.

**Electronic supplementary material:**

The online version of this article (doi:10.1186/s12864-015-1785-9) contains supplementary material, which is available to authorized users.

## Background

Large amounts of microarray experimental data are available in public repositories. Although a variety of programs have been developed to make use of these data, the number of tools that identify marker genes is limited. Genes may be split into two categories based on the number of tissues in which they are expressed [1]. Genes that are expressed in many tissues are often designated as housekeeping while those that are expressed in few tissues are termed tissue-specific or marker genes. Marker genes are used to determine the tissue identity and to characterize cells grown *in vitro*.

Since disease-associated genes are more likely to show tissue specific expression [2], marker genes of healthy tissues could also be used to understand the molecular mechanisms underlying complex diseases.

Microarrays allow the parallel analysis of the expression of several thousands of genes in hundreds of tissues/cell types, and have been extensively used by the scientific community. Consequently, a large amount of microarray expression data has accumulated in public repositories. The Gene Expression Omnibus (GEO) [3], contains currently expression data on 1,328,979 samples across 3848 datasets, and ArrayExpress [4] contains 1,649,790 assays across 55,656 experiments. The aim of the current study was to develop a tool to detect marker genes associated with small sets of normal tissue samples obtained from microarray experiments. Here we introduce a new computational tool to predict marker genes from microarray gene expression data. The tool is available as a stand-alone version (R-package called MGFM) in Bioconductor [5] and it is also integrated into the CellFinder platform (http://cellfinder.org/analysis/marker) to be used as an online tool. CellFinder [6] is a comprehensive one-stop resource for diverse data characterizing mammalian cells in different tissues and in different development stages. It is built from carefully selected data sets stemming from other curated databases and the biomedical literature.

We verified MGFM using two microarray data sets from the GEO public repository each encompassing samples for similar sets of five human tissues (brain, heart, kidney, liver, and lung). The accuracy of MGFM was verified with known literature-curated marker genes. Using one of the data sets MGFM identified 72 % of the known marker genes. Moreover, top ranked marker genes were further validated by RT-PCR.

## Results

**Fig. 1:**
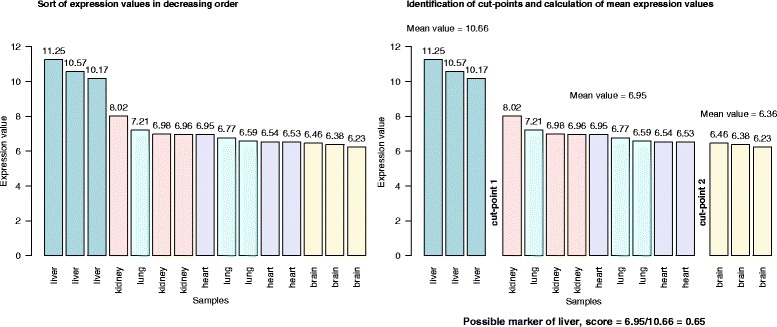
An example showing how marker genes are identified by our method. The expression values correspond to the probe set *"*202357_*s*_*a* *t* *"*, which represents the gene *CFB* (complement factor B)

Marker genes are identified when sample grouping of the same type exist with similar expression of the marker gene (see Fig. 1 for an illustrative example and Materials and methods for details). After sorting the expression values of probe sets in decreasing order, a probe set is considered a potential marker of a sample type if the highest expression values represent all replicates of this sample type. We consider the position in the sorted expression vector that segregates different sample types a cut-point. Each cut-point segregates elements of sample types into two distinct sample groups. For each probe set, the expression levels of the two sample groups are summarized as the mean of expression values. The marker genes can then be ranked according to a score ranging from 0 to 1, which is the ratio of the second and first value in the vector of mean expression values of a probe set. Values near 0 would indicate high specificity and large values closer to 1 would indicate low specificity.

We applied MGFM to two microarray data sets from GEO. The first data set (#1) consists of triplicate samples from five human tissues (heart atrium, kidney cortex, liver, lung, and midbrain). The microarray data set is publicly available from GEO with the series number GSE3526 [7]. The second data set (#2) is derived from five human tissues (brain, heart, kidney, liver, and lung) from two GEO Series GSE1133 [8] and GSE2361 [9] (see Table 7). Moreover, we compared the results with another tool for tissue-specific gene identification [10] and validated the identified markers using literature-curated markers (Additional file 1) and experimentally by RT-PCR.

### Marker selection

**Fig. 2:**
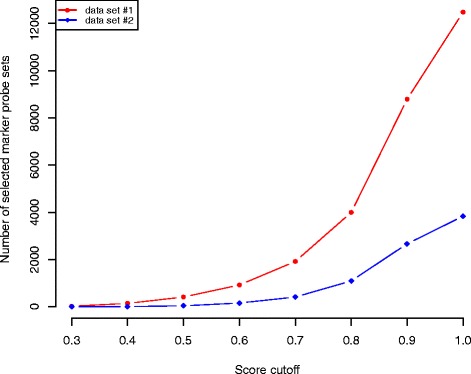
Number of marker probe sets found for each cutoff for data sets #1 and #2

For data set #1, 12482 probe sets out of 54675 (comprising about 23 % of all probe sets on the microarray) were identified as potential markers associated with the five analyzed tissues. In data set #2 we identified 3836 probe sets from 22283 as potential markers, or approximately 17 % of the probes on the microarray. In order to refine the number of predicted markers, they were ranked according to their score (see Materials and methods for details, Identification of marker genes). To investigate how the number of selected markers changes depending on the score, we used different cutoffs (Fig. 2). The number of potential markers selected increases with less specific (higher) cutoffs.

### Performance analysis

**Fig. 3:**
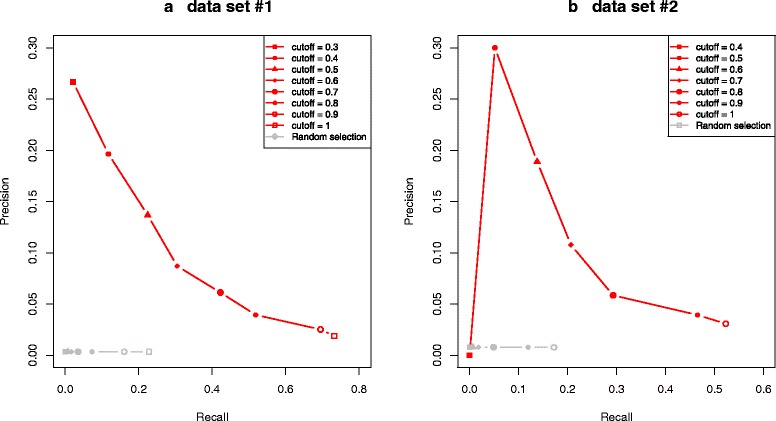
Precision/Recall curves for the complete set of genes selected by MGFM as potential markers for the examined tissues using **a** data set #1 and **b** data set #2. The gray curves show precision/recall for random selection

**Fig. 4:**
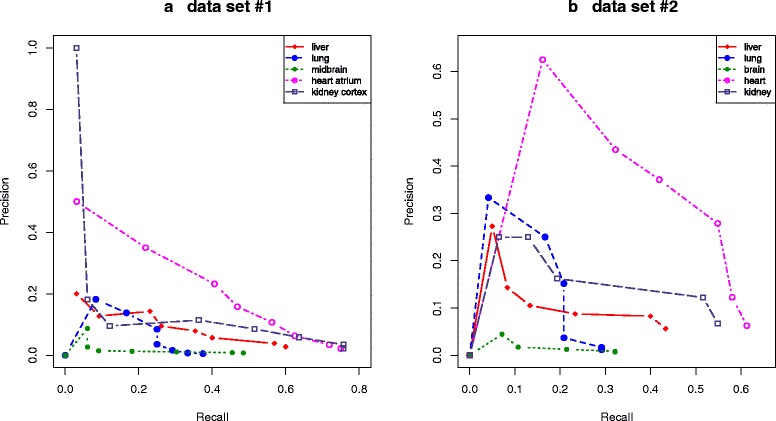
Precision/Recall curves for genes selected by MGFM as potential markers for each of the examined tissues using **a** data set #1 and **b** data set #2

To evaluate the precision of the developed tool, we searched the literature to collect genes used extensively as markers for cell types within a tissue. A total of 142 literature-derived genes were found for the five human tissues (brain, heart, kidney, liver, and lung) and will here be called real markers (Additional file 1). In addition to these markers, the cytochrome P450 genes (51 genes) were used as markers for liver, since these genes are highly expressed in the liver [11]. For validation of our potential marker sets, only real marker genes that were also found on the microarray of each data set were considered for the validation. This corresponds to 187 marker genes for data set #1 and 174 for data set #2. To validate the performance of MGFM, the recall and precision were examined using the collected markers. Two strategies were used: i) The predicted markers for each of the examined tissues were combined and compared with the complete set of known markers of all examined tissues. ii) The set of predicted markers for each tissue was compared with the known markers of this tissue. Recall and precision were analyzed, where recall is the fraction of identified marker genes in the total number of real markers and precision is the fraction of marker genes identified in the total number of predicted marker genes. Figures 3a) and 3b) show the precision/recall curves for marker genes predicted by MGFM using data set #1 and data set #2, respectively. The grey curves show the precision/recall for random selection. As illustrated, MGFM performed better than random selection in both data sets. Using lower score cutoffs results in higher precision and lower recall, whereas higher score cutoffs results in lower precision and higher recall. Tables 1 and 2 show the percentage of probes on the microarray predicted as marker probe sets and the percentage of correctly identified marker genes using different score cutoffs for data sets #1 and #2, respectively (see Materials and methods for details on how probe sets were mapped to genes). Decreasing the score from 1 to 0.9 reduced the percentage of probe sets predicted as markers from 22.8 % (of 54675 probes on the microarray) to 16 % (minus 6.8 %), while losing only 3.8 % of the known literature-collected markers (see Table 1). Using data set #2, MGFM predicted 17 % of the probes on the microarray (22283 probe sets) as potential markers for the examined tissues, which contain approximately 52 % of the real markers. In comparison to the results achieved by applying MGFM to data set #1, the reduction was higher, while the precision was lower. Figures 4a) and 4b) show the precision/recall curves for the predicted marker genes of the examined tissues in data sets #1 and #2, respectively. In both data sets the performance of MGFM differs depending on the tissues. The best performance is achieved for heart or heart atrium, whereas the lowest precision was obtained for brain or midbrain. Table 3 shows the number of correctly identified and known marker genes on the microarrays of data sets #1 and #2 for each of the examined tissues.

**Table 1:** The percentage of probes on the microarray predicted as marker probe sets and the percentage of correctly identified marker genes using different score cutoffs for data set #1

Score cutoff	1	0.9	0.8	0.7	0.6	0.5	0.4	0.3
Selected marker probe sets (in %)	22.8	16	7.3	3.5	1.7	0.7	0.3	0.04
Identified marker genes (in %)	72.2	68.4	51.9	42.2	30.5	22.5	11.8	2.1

**Table 2:** The percentage of probes on the microarray predicted as marker probe sets and the percentage of correctly identified marker genes using different score cutoffs for data set #2

Score cutoff	1	0.9	0.8	0.7	0.6	0.5	0.4
Selected marker probe sets (in %)	17.2	11.9	4.9	1.8	0.7	0.2	0.02
Identified marker genes (in %)	51.7	46	29.3	20.7	13.8	5.2	0

**Table 3:** The number of correctly identified and known marker genes on the microarrays of data sets #1 and #2 for each of the examined tissues

	Tissue	Correctly identified/known
		marker genes on the microarray
Data	midbrain	16/33
set	heart atrium	24/32
#1	kidney cortex	25/33
	liver	39/65
	lung	9/24
Data	liver	26/60
set	lung	7/24
#2	brain	9/28
	kidney	17/31
	heart	19/31

### Comparison to *t*-test

A possible method to identify marker gene candidates is to identify genes that are differentially expressed between two experimental groups using a statistical test such as a *t*-test. Genes associated with each sample type could be used as markers. In order to further verify the performance of our method, we applied *t*-test to both data sets #1 and #2. Each tissue was compared against the other tissues. The predicted markers for each of the examined tissues were combined and compared with the complete set of known markers of all examined tissues. At a score cutoff of 0.9 MGFM outperformed the *t*-test (*p* value range: from 0.01 to 0.09) in terms of precision (see Additional file 2: Figures S1 and S2).

### Overlap of sets of predicted marker genes

**Fig. 5:**
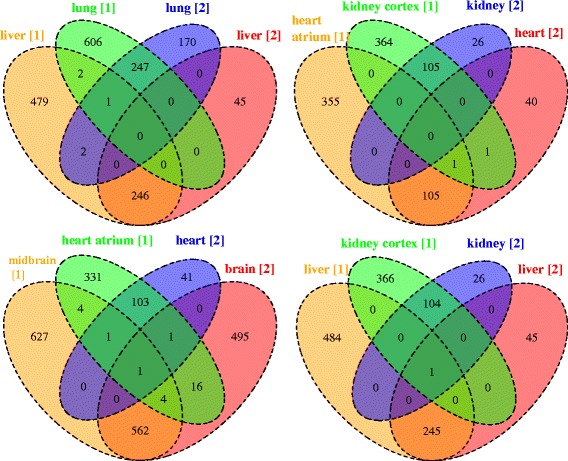
Venn diagrams showing comparisons of the predicted marker gene lists for the examined tissues. Labels in the Venn diagrams indicate tissue and data set (1 or 2, within brackets)

Next we compared the results obtained using data sets #1 and #2. The aim was to confirm that the selection of marker genes by MGFM was consistent with the tissues analyzed even if the data compared was obtained from different platforms: Affymetrix Human Genome U133A Array (GPL96) and Affymetrix Human Genome U133 Plus 2.0 Array (GPL570), for data sets #1 and #2, respectively. Figure 5 shows Venn diagrams that illustrate comparisons of the predicted marker gene lists for the examined tissues using both data sets #1 and #2. Obviously, the overlap of marker genes for the same tissue is significantly higher than the overlap of markers for different tissues. These results suggest a possible strategy to reduce the false positives by applying MGFM to more than one data set including the tissue of interest, and to consider the intersection of sets of markers associated with the tissue of interest.

### Enrichment of Gene Ontology terms

To assess whether the subsets of marker genes show significant over-representation of biological characteristics related to their corresponding tissues, Gene Ontology (GO) [12] enrichment analysis was performed. Tables 4 and 5 show the enriched molecular function and the enriched biological process of markers associated with the examined tissues using data set #1 at a score cutoff of 0.9. For each tissue five significantly enriched GO terms that do not overlap more than 80 % are displayed. In the case of molecular functions, we remark *tropomyosin binding* and *actin binding* for heart (because of the heart muscle), *antiporter activity* for the kidney, *receptor binding* for the lung, and *GTP binding* for the midbrain (signal transduction). With respect to the biological process, we remark *xenobiotic metabolic process* for the liver, *transmembrane transport* for the kidney (salt and water transport), and *neurotransmitter transport* or *regulation of transmission of nerve impulse* for the midbrain.

**Table 4:** Gene Ontology enrichment (Molecular Function) of predicted marker genes for the examined tissues

GO ID	GO	*p*-value	Expected count	Gene count	Size
Midbrain					
GO:0008017	microtubule binding	1.02×10^−11^	14.02	43	148
GO:0030695	GTPase regulator activity	2.51×10^−07^	39.97	73	422
GO:0005525	GTP binding	4.18×10^−06^	31.73	58	335
GO:0030276	clathrin binding	4.27×10^−06^	1.89	10	20
GO:0017075	syntaxin-1 binding	5.28×10^−06^	1.23	8	13
Heart atrium					
GO:0008307	structural constituent of muscle	3.12×10^−24^	1.73	25	44
GO:0003779	actin binding	2.81×10^−21^	13.63	58	346
GO:0005523	tropomyosin binding	1.34×10^−08^	0.55	8	14
GO:0051371	muscle alpha-actinin binding	2.46×10^−08^	0.28	6	7
GO:0031432	titin binding	4.07×10^−08^	0.43	7	11
Kidney cortex					
GO:0008509	anion transmembrane transporter activity	4.9×10^−16^	8.77	40	226
GO:0015294	solute:cation symporter activity	8.98×10^−11^	3.03	19	78
GO:0015081	sodium ion transmembrane transporter activity	4.5×10^−09^	4.58	21	118
GO:0015297	antiporter activity	1.92×10^−08^	2.17	14	56
GO:0019534	toxin transporter activity	1.39×10^−04^	0.31	4	8
Liver					
GO:0004497	monooxygenase activity	2.23×10^−20^	4.95	34	87
GO:0009055	electron carrier activity	4×10^−20^	8.20	43	144
GO:0048037	cofactor binding	1.17×10^−16^	14.11	52	248
GO:0020037	heme binding	2.06×10^−15^	6.83	34	120
GO:0005506	iron ion binding	1.53×10^−14^	8.54	37	150
Lung					
GO:0005102	receptor binding	3.30×10^−10^	71.09	124	1129
GO:0004896	cytokine receptor activity	5.12×10^−09^	5.23	22	83
GO:0003823	antigen binding	1.78×10^−08^	3.02	16	48
GO:0019899	enzyme binding	6.89×10^−07^	69.58	110	1105
GO:0032395	MHC class II receptor activity	1.54×10^−06^	0.5	6	8

Column labels are as follows: GO ID is the GO identifier; GO is the description of the GO term; *p*-value is the hypergeometric *p*-value for over-representation of each GO term; Expected/Gene Count are the expected and actual gene counts; and Size is the number of genes within each GO term

**Table 5:** Gene Ontology enrichment (Biological Process) of predicted marker genes for the examined tissues

GO ID	GO	*p*-value	Expected count	Gene count	Size
Midbrain					
GO:0007409	axonogenesis	4.49×10^−22^	46.71	118	489
GO:0010975	regulation of neuron projection development	5.58×10^−20^	21.4	70	224
GO:0006836	neurotransmitter transport	3.93×10^−18^	12.42	49	130
GO:0051969	regulation of transmission of nerve impulse	9.65×10^−16^	19.11	59	200
GO:0016358	dendrite development	6.24×10^−13^	12.51	42	131
Heart atrium					
GO:0006941	striated muscle contraction	3.12×10^−27^	3.82	37	97
GO:0060047	heart contraction	3.34×10^−27^	5.76	44	146
GO:0048738	cardiac muscle tissue development	9.63×10^−25^	5.56	41	141
GO:0090257	regulation of muscle system process	4.29×10^−24^	5.76	41	146
GO:0030239	myofibril assembly	1.6×10^−26^	1.66	26	42
Kidney cortex					
GO:0055085	transmembrane transport	2.03×10^−18^	29.19	83	757
GO:0007588	excretion	1.83×10^−10^	2.47	17	64
GO:0072006	nephron development	6.35×10^−09^	3.43	18	89
GO:0006814	sodium ion transport	8.87×10^−08^	4.47	19	116
GO:0072348	sulfur compound transport	1.74×10^−05^	0.89	7	23
Liver					
GO:0008202	steroid metabolic process	8.68×10^−46^	15.33	90	267
GO:0032787	monocarboxylic acid metabolic process	3.34×10^−35^	24.57	100	428
GO:0006805	xenobiotic metabolic process	4.1×10^−30^	8.04	53	140
GO:0044282	small molecule catabolic process	2×10^−28^	14.7	69	256
GO:1901605	alpha-amino acid metabolic process	7.38×10^−25^	11.54	57	201
Lung					
GO:0002684	positive regulation of immune system process	2.53×10^−37^	40.21	134	606
GO:0006954	inflammatory response	2.23×10^−25^	32.64	101	492
GO:0001816	cytokine production	4.95×10^−25^	31.32	98	472
GO:0046649	lymphocyte activation	1.2×10^−24^	31.12	97	469
GO:0009607	response to biotic stimulus	2.18×10^−24^	39.28	111	592

Column labels are as follows: GO ID is the GO identifier; GO is the description of the GO term; *p*-value is the hypergeometric *p*-value for over-representation of each GO term; Expected/Gene Count are the expected and actual gene counts; and Size is the number of genes within each GO term

### PCR analysis

To verify the tissue-specific expression of top-ranked marker genes, we examined these genes by RT-PCR. Top ranked marker genes predicted using both data sets #1 and #2 were investigated. A total of 11 marker genes were selected for liver and 12 genes for each of the tissues: brain, heart, kidney, and lung. The resulting gel electrophoresis images are shown in Additional file 3: Figures S1, S2, S3, S4, S5, S6, S7, S8, S9, S10 and S11. In addition, the PCR results are summarized in Table 6 using + or - for present or absent, respectively. As shown in Table 6, all genes, predicted as markers of a tissue, were indeed detected in that tissue except *GAP43* in the brain, and the four genes *SLC12A1*, *SLC3A1*, *FXYD2*, and *CA12* predicted as markers of kidney.

**Table 6:** PCR results

Predicted marker genes for liver											
Gene	Liver	Lung	Heart	Brain	Kidney	Gene	Liver	Lung	Heart	Brain	Kidney
*AKR1D1*	+	+	-	-	-	*CYP2E1*	+	-	-	-	-
*FGG*	+	-	+	-	-	*APOC3*	+	-	-	-	-
*APOA2*	+	+	-	-	-	*SERPINC1*	+	-	-	-	-
*CYP2C8*	+	-	-	-	-	*AHSG*	+	-	-	-	-
*GC*	+	-	-	-	-	*AMBP*	+	-	-	-	-
*CPS1*	+	-	-	-	-						
Predicted marker genes for lung											
Gene	Liver	Lung	Heart	Brain	Kidney	Gene	Liver	Lung	Heart	Brain	Kidney
*CLDN18*	-	+	-	-	-	*LAMP3*	-	+	+	-	-
*NKX2-1*	-	+	+	-	-	*AGER*	-	+	-	-	-
*SCGB1A1*	-	+	+	-	-	*LYZ*	+	+	+	-	-
*SFTPB*	-	+	-	-	-	*SFTPD*	-	+	-	-	-
*CYP4B1*	-	+	+	-	-	*SFTPC*	-	+	-	-	-
*CD52*	-	+	+	-	-	*SLC34A2*	-	+	-	-	+
Predicted marker genes for heart											
Gene	Liver	Lung	Heart	Brain	Kidney	Gene	Liver	Lung	Heart	Brain	Kidney
*MYOZ2*	-	-	+	-	-	*PLN*	-	+	+	-	+
*TNNI3*	-	+	+	-	-	*MB*	-	-	+	-	-
*SYNPO2L*	-	+	+	-	-	*TTN*	-	+	+	-	+
*MYH6*	-	-	+	-	-	*MYL7*	-	-	+	-	-
*CSRP3*	-	-	+	-	-	*MYH7*	-	-	+	-	-
*CKM*	-	-	+	-	-	*TPM1*	+	+	+	-	+
Predicted marker genes for brain											
Gene	Liver	Lung	Heart	Brain	Kidney	Gene	Liver	Lung	Heart	Brain	Kidney
*GAP43*	-	-	-	-	-	*MBP*	-	+	+	+	+
*GFAP*	-	-	-	+	-	*GRIA2*	-	-	-	+	-
*TMEFF1*	-	-	-	+	-	*KIF5C*	-	-	-	+	-
*FUT9*	-	-	-	+	+	*STMN2*	-	-	-	+	-
*SYT1*	-	-	-	+	-	*NEFM*	-	-	-	+	-
*SNAP25*	-	+	+	+	-	*GABBR2*	-	-	-	+	-
Predicted marker genes for kidney											
Gene	Liver	Lung	Heart	Brain	Kidney	Gene	Liver	Lung	Heart	Brain	Kidney
*SLC12A1*	-	-	-	-	-	*CA12*	-	-	-	-	-
*SLC3A1*	-	-	-	-	-	*PDZK1IP1*	-	-	-	-	+
*UMOD*	-	-	-	-	+	*FXYD2*	-	-	-	-	-
*AOC1*	-	-	-	-	+	*CDH16*	-	-	-	-	+
*CD24*	-	+	-	-	+	*SLC22A8*	-	-	-	-	+
*HSD11B2*	-	+	-	-	+	*CLDN8*	-	-	-	-	+

### Detection of novel marker genes

All identified marker genes are shown in Additional file 4 and descriptions of their functions provided if available. There are 11 liver specific genes predicted and 12 genes for each of the other four tissues. The set of marker genes predicted by MGFM contained genes that have been recently reported as novel marker genes, such as *SYNPO2L* in the heart, *KIF5C* in the brain and *AMDHD1* in the liver. *SYNPO2L* encodes a cytoskeletal protein. Beqqali et al. [13] recently reported the corresponding protein as a novel protein that interacts and colocalizes with *α*-actinin at the Z-disc of the sarcomere. In a recent study, Willemsen et al. [14] suggested that mutations in *KIF4A* and *KIF5C* cause intellectual disability by tipping the balance between excitatory and inhibitory synaptic excitability. These results indicate a role of *KIF5C* in brain function. Song et al. [10] reported *AMDHD1* as new marker for liver. Hence, our comparatively easily implementable method was able to discover novel marker genes.

## Discussion

In this work, we presented a new tool for detection of marker genes from microarray gene expression data. The tool is provided as a standalone version (a Bioconductor package called MGFM) as well as a web application within the CellFinder platform.

Using two different data sets, at a score cutoff of 0.9, MGFM validated 68.4 % of literature-curated markers while reducing the total number of probe sets predicted as markers from 54675 to 8789 (approximately 16 % of the probes on the microarray) and validated 46 % of literature-curated real marker genes while reducing the total number of predicted marker probe sets from 22283 to 2664 (approximately 11.9 % of the probes), respectively.

Song et al. [10] developed a method to identify tissue-specific genes by analyzing microarray data. They used the GEO data set GDS596 (see Table 7, data set #3) to identify marker genes for the tissues: fat, heart, kidney, liver, and lung. Song et al. reported that they confirmed 10 kidney, 11 liver, 11 lung, and 11 heart marker genes by applying their approach. To assess if we would find these genes using MGFM, we applied it to the same data set using the samples representing the tissues: heart, kidney, liver, and lung. All of these genes were found as potential markers by MGFM except the genes *AMDHD1* (amidohydrolase domain containing 1) for liver and *PRUNE2* (prune homolog 2) for heart. Song et al. reported these two genes as new markers. We investigated if these genes were found by MGFM using data sets #1 and #2. The gene *AMDHD1* was predicted by MGFM as potential marker for liver using data set #1. The gene *PRUNE2* was predicted by MGFM as marker for brain or midbrain using both data sets #1 and #2. Song et al. described their method but did not provide a tool for use. Here, we provide a tool implemented in the R programming language that can be easily used by calling the appropriate functions. Finally, we were able to verify the marker genes experimentally by comparative PCR in all five tissues. While not all marker genes were unambiguous markers, and some were not detected, the vast majority (92 %) was experimentally confirmed (Table 6).

**Table 7:** The corresponding samples to the tissues in the 3 data sets

	Tissue	Samples
Data	midbrain	GSM80699, GSM80700, GSM80701
set	heart atrium	GSM80654, GSM80655, GSM80656
#1	kidney cortex	GSM80686, GSM80687, GSM80688
	liver	GSM80728, GSM80729, GSM80730
	lung	GSM80707, GSM80710, GSM80712
Data	liver	GSM44702, GSM18953, GSM18954
set	lung	GSM44704, GSM18949, GSM18950
#2	brain	GSM44690, GSM18921, GSM18922
	kidney	GSM44675, GSM18955, GSM18956
	heart	GSM44671, GSM18951, GSM18952
Data	liver	GSM18953, GSM18954
set	lung	GSM18949, GSM18950
#3	heart	GSM18951, GSM18952
	kidney	GSM18955, GSM18956

A description of the different marker genes identified by MGFM is provided in Additional file 4.

### Uses of MGFM in CellFinder

To date, MGFM can be used within CellFinder for the data sets applied in the current study and will be extended by storing preprocessed expression data derived from different tissues and cell types to enable the identification of marker genes associated with a set of tissue samples or cell types in an easy, fast and accurate way. More specifically, MGFM has the following features to i) allow users to conveniently modify the set of samples of interest by adding or removing samples, ii) calculate the potential marker genes at the gene level (using JetSet [15] to associate genes to probe sets), iii) display and rank the list of marker genes associated with each sample type according to the specificity, and iv) download the list of all found markers for further use. Moreover, probe sets are linked to CellFinder’s gene view which allows for an immediate evaluation of potential marker genes utilizing expression values from the RNA Seq Atlas [16]. Also, gene ontology annotations [12] are included for better understanding of functional properties of genes.

## Conclusion

We presented a new method for marker gene detection using microarray gene expression data. We verified this method using two data sets from GEO describing gene expression in comparable sets of five human tissues. The sets of predicted marker genes associated with the examined tissues comprised several well-known genes of particular importance in these tissues. Furthermore, we confirmed the tissue specific expression of predicted novel markers by RT-PCR.

In summary, the main advantages of the application presented herein are i) a short running time of some seconds per analysis. This is achieved by sorting the gene expression values instead of using gene differential expression. ii) MGFM offers the user the possibility to modify the set of samples by easily removing or adding new samples. iii) MGFM is available as a standalone version (R-package) as well as a web application integrated into the CellFinder platform. We are currently working on a database to store preprocessed expression data derived from different tissues and cell types, in order to enable the identification of marker genes associated with a set of samples of interest in a convenient and fast way.

## Materials and methods

### Data sources

The microarray expression data are derived from GEO. The first data set (#1) consists of 15 samples and is derived from five human tissues (heart atrium, kidney cortex, liver, lung, and midbrain). The microarray data set is publicly available from GEO with the series number GSE3526 [7]. The second data set (#2) is derived from five human tissues (brain, heart, kidney, liver, and lung) from two GEO Series GSE1133 [8] and GSE2361 [9]. The third data set (#3) (used by Song et al. [10]) is derived from four human tissues (heart, kidney, liver, and lung) from the GEO DataSet GDS596. Each tissue is represented by two to three samples. Table 7 shows the samples that represent the tissues in the three data sets.

### Data normalization

The Robust Multiarray Averaging [17] (RMA) procedure was used for background correction, normalization, and summarization of the AffyBatch probe-level data for data sets #1 and #2. In addition, data set #2 was normalized using the ComBat method from the R-package sva (Version: 3.6.0) [18] in order to remove batch effects.

### Identification of marker genes

Marker genes are identified following the steps below: **Sort of expression values for each probe set:** In this step the expression values are sorted in decreasing order.**Marker selection**: To analyze the sorted distribution of expression values of a probe set to define if it is a potential candidate marker we define cut-points as those that segregate samples of different types. A sorted distribution can have multiple cut-points; a cut-point can segregate one sample type from the others, or it can segregate multiple sample types from multiple sample types. In the example given in Fig. 1, the distribution has two cut-points (cut-point 1 and cut-point 2), the first cut-point segregates liver samples from the rest, and the second cut-point segregates brain samples from the rest. Each cut-point is defined by the ratio of the expression averages of the groups of samples adjacent to it. That is, given a distribution with n cut-points and n+1 segregated groups, cut-point i receives a score that is the ratio of the average expression of samples in the group i+1 (following the cut-point) divided by that of group i (preceding the cut-point). This value is < 1 because the values are sorted in decreasing order. The closer the values, the closer the score to 1 and therefore the smaller is the gap between expression values at the cut-point. We use this score to indicate the specificity of the cut-point and by extension of the probe set as marker between particular groups of tissues. For simplicity, in this work we take only probe sets as markers if they have a cut-point that segregates one tissue at high expression from the rest (as in Fig. 1 for liver). We disregard negative markers (segregating samples from one tissue at low expression) or multiple tissue markers (segregating samples from more than one tissue from other multiple tissues). However, our method can calculate them (for example, as in Fig. 1, *CFB* can be defined as a positive marker for liver and as a negative marker for brain).**Mapping of probe sets to genes:** Affymetrix probe sets were mapped to Entrez GeneIDs using the 23 October 2013 release of NetAffx annotations [19].

### Calculation of precision/recall curves

To validate the performance of MGFM, the recall and precision were examined using the literature-curated known markers. Two strategies were used: i) The predicted markers for each of the examined tissues were combined and compared with the complete set of known markers of all examined tissues. ii) The set of predicted markers for each tissue was compared with the known markers of this tissue. A marker gene is considered as identified if the corresponding selected probe set maps unambiguously to this gene.

### Gene Ontology enrichment analysis

Gene ontology enrichment analysis was assessed with the hypergeometric statistic as implemented in the R-package GOstats [20] (Version: 2.32.0), with all genes on the microarray as background. The cutoff for *p*-values was 0.01.

### Venn diagrams

The Venn diagrams were generated using the R-package VennDiagram (Version: 1.6.0) [21].

### *t*-test

The *p*-values were adjusted for multiple testing using the Benjamini-Hochberg procedure.

### Ethics statement

Human kidney tissue was provided from leftover diagnostic biopsies from the Department of Nephrology at Charite Universitätsmedizin Berlin. RNA from heart and lung tissues was provided by the German Heart Center Berlin, and RNA from liver from the Department of Experimenal Surgery at Charite Universitätsmedizin Berlin. All tissue donors were consented and ethics approval obtained from the responsible ethics Committee at Charite (Nr. EA1/110/10) and the German Heart Center (Nr. EA4/028/12).

### cDNA synthesis and PCR analysis

Human total RNA was isolated from liver, lung, heart and kidney with TRIzol reagent (Invitrogen) according to the manufacturer’s protocol. Human RNA from brain was purchased from Clontech Laboratories (Mountain View, CA, USA). RNA was reverse transcribed into cDNA with random primers using SuperScript III First-Strand Synthesis System (Invitrogen) according to the manufacturer’s protocol. Five *μ*g of total RNA was used for cDNA synthesis.

The PCR reaction consisted of 1 *μ*l of cDNA, 0.5 *μ*l of 10 mM deoxynucleoside triphosphate mix (dNTP), 5 *μ*l of 5X Crimson Taq (Mg-free) Reaction Buffer, 1.5 *μ*l of 25 mM MgCl _2_, 0.5 *μ*l of each 10 *μ*M forward and reverse primers, 0.125 *μ*l of Crimson Taq DNA polymerase, and nuclease-free water up to 25 *μ*l. The cycling conditions were performed as following: 95 °C for 2 min, followed by 30 cycles of 95 °C for 30 s, temperature specific annealing for 30 s, and 72 °C for 45 s with a final elongation at 72 °C for 7 min. A 1 % agarose gel was used to check PCR amplification. All primers used are listed in Additional file 5. The housekeeping gene beta-actin was used as positive control.

### Tool requirements

MGFM expects replicates for each sample type. Using replicates has the advantage of increased precision of gene expression measurements and allows smaller changes to be detected. It is not necessary to use the same number of replicates for all sample types. Normalization is necessary before any analysis to ensure that differences in intensities are indeed due to differential expression, and not to some experimental factors that add systematic biases to the measurements. Hence, for reliable results normalization of data is mandatory. When combining data from different studies, other procedures should be applied to adjust for batch effects.

### Implementation of the online tool

The online version of MGFM integrated into CellFinder is implemented in JavaScript for the frontend and PHP together with Rserve [22] for the backend. JavaScript is utilized to allow for asynchronous user interactions and requests are sent to a PHP webservice, which handles in and outputs and controls Rserve to call MGFM.

### Software availability

The R-package MGFM is freely available from the Bioconductor web site (http://www.bioconductor.org/packages/release/bioc/html/MGFM.html).

## Additional files

Additional file 1
**Literature-curated marker genes.** This file includes marker genes collected from the literature. (104KB PDF)

Additional file 2
**Plots of Precision/Recall comparing our method to** ***t*** **-test.** This file includes Plots of Precision/Recall comparing MGFM to *t*-test. (462KB PDF)

Additional file 3
**Gel electrophoresis images.** This file includes the gel electrophoresis images (Figures S1–S11). (981KB PDF)

Additional file 4
**Description of the predicted marker genes.** (126KB PDF)

Additional file 5
**Primer sequences.** This file includes the list of all primer sequences used by PCR. (55.7KB PDF)

## Abbreviations

GEO: Gene expression omnibus
RT-PCR: Reverse transcriptase-polymerase chain reaction
MGFM: Marker gene finder in microarray data
GO: Gene ontology
