# Supplementary material for: MGFM: a novel tool for detection of tissue and cell specific marker genes from microarray gene expression data
Source: BMC Genomics. 2015 Aug 28;16(1):645. doi: 10.1186/s12864-015-1785-9 (PMC4552366; doi:10.1186/s12864-015-1785-9)
Supplement: Additional file 1 — Literature-curated marker genes. This file includes marker genes collected from the literature. (104KB PDF) [file 12864_2015_1785_MOESM1_ESM.pdf]

Literature-curated marker genes

References

[1] Lan, X., Rai, P., Chandel, N., Cheng, K., Lederman, R., Saleem, M.a., Mathieson, P.W., Husain, M., Crosson, J.T., Gupta, K., Malhotra, A., Singhal, P.C.: Morphine induces albuminuria by compromising podocyte integrity. PloS one **8**(3), 55748 (2013). doi:10.1371/journal.pone.0055748

[2] Kelder, T.P., Penning, M.E., Uh, H.-W., Cohen, D., Bloemenkamp, K.W.M., Bruijn, J.a., Scherjon, S.a., Baelde, H.J.: Quantitative polymerase chain reaction-based analysis of podocyturia is a feasible diagnostic tool in preeclampsia. Hypertension **60**(6), 1538–1544 (2012). doi:10.1161/HYPERTENSIONAHA.112.201681

[3] Song, B., Smink, A.M., Jones, C.V., Callaghan, J.M., Firth, S.D., Bernard, C.a., Laslett, A.L., Kerr, P.G., Ricardo, S.D.: The directed differentiation of human iPS cells into kidney podocytes. PloS one **7**(9), 46453 (2012). doi:10.1371/journal.pone.0046453

[4] Hill, G.S., Karoui, K.E., Karras, A., Mandet, C., Duong Van Huyen, J.-P., Nochy, D., Bruneval, P.: Focal segmental glomerulosclerosis plays a major role in the progression of IgA nephropathy. I. Immunohistochemical studies. Kidney international **79**(6), 635–642 (2011). doi:10.1038/ki.2010.466

[5] Lindgren, D., Boström, A.-K., Nilsson, K., Hansson, J., Sjölund, J., Möller, C., Jirström, K., Nilsson, E., Landberg, G., Axelson, H.k., Johansson, M.E.: Isolation and characterization of progenitor-like cells from human renal proximal tubules. The American journal of pathology **178**(2), 828–837 (2011). doi:10.1016/j.ajpath.2010.10.026

[6] Little, M.H., Bertram, J.F.: Is there such a thing as a renal stem cell? Journal of the American Society of Nephrology : JASN **20**(10), 2112–2117 (2009). doi:10.1681/ASN.2009010066

[7] Jászai, J., Farkas, L.M., Fargeas, C.a., Janich, P., Haase, M., Huttner, W.B., Corbeil, D.: Prominin-2 is a novel marker of distal tubules and collecting ducts of the human and murine kidney. Histochemistry and cell biology **133**(5), 527–539 (2010). doi:10.1007/s00418-010-0690-1

[8] Damaraju, V.L., Elwi, A.N., Hunter, C., Carpenter, P., Santos, C., Barron, G.M., Sun, X., Baldwin, S.A., Young, J.D., Mackey, J.R., Sawyer, M.B., Cass, C.E.: Localization of broadly selective equilibrative and concentrative nucleoside transporters , hENT1 and hCNT3 , in human kidney Localization of broadly selective equilibrative and concentrative nucleoside transporters , hENT1 and hCNT3 , in human kidney (April 2007) (2013). doi:10.1152/ajprenal.00007.2007

Literature-curated marker genes

kidney	Reference	kidney	Reference
NPHS1	[1], [2], [3]	*NPHS2*	[1], [2], [3]
SYNPO	[1], [3]	*WT1*	[1], [3], [4]
CD2AP	[1]	*VEGF*	[2], [4]
PAX2	[3]	*PROM1*	[5]
CD24	[5]	*AQP1*	[6]
AQP2	[6]	*CLCNKB*	[6]
PROM2	[7]	*UMOD*	[8]
PTPRO	[4]	*ANPEP*	[9]
CYP27B1	[9]	*LRP2*	[9]
ATP1A1	[9]	*SLC2A1*	[9]
SLC5A10	[9]	*SLC4A4*	[9]
SLCO1B1	[9]	*SLC22A8*	[9]
POU2F1	[9]	*SLC22A5*	[9]
ABCB1	[9]	*SLC15A1*	[9]
SLC15A2	[9]	*SLC12A3*	[9]
SLC12A1	[9]	*PODXL*	[9]
AQP3	[9]	*ACE2*	[10]

heart	Reference	heart	Reference
GJA1	[11]	*MYPN*	[11]
TNNI3	[11]	*TNNT2*	[11]
DES	[11]	*MYH7*	[11]
ACTC1	[11]	*NKX2-5*	[12]
TPM1	[12]	*ACTN1*	[12]
TPM2	[12]	*TPM3*	[12]
TPM4	[12]	*MYL12B*	[13]
ACTN2	[12]	*ACTN3*	[12]
ACTN4	[12]	*DDR2*	[12]
NPPA	[14]	*NPPB*	[15]
ANKRD1	[16]	*CAV2*	[17]
CAV3	[18]	*MYL7*	[13]
HAND1	[19]	*HAND2*	[20]
GATA4	[19]	*MYH*	[21]
MYL2	[22]	*FHL2*	[23]
HSPB7	[24]	*MYOZ2*	[25]

lung	Reference	lung	Reference
SFTPD	[26]	*MUC1*	[26]
SFTPC	[27]	*LAMP3*	[28]
THY1	[29]	*AQP5*	[29]
PGC	[30]	*ICAM1*	[30]
CLDN7	[30]	*SERPINE1*	[30]
ABCA1	[31]	*CCL2*	[32]
CCR2	[32]	*GNB2L1*	[33]
MME	[34]	*TUBB4A*	[35]
KCNA5	[36]	*CSF1R*	[37]
TMPRSS11D	[38]	*SCGB1A1*	[39]
PDPN	[40]	*FOXJ1*	[40]
TTF1	[40]	*KRT14*	[41]
SLURP1	[42]		

liver	Reference	liver	Reference
ALB	[43],[44]	*DPP4*	[43],[45]
CYP3A4	[43]	*PCK1CPS1*	[43]
CPS1	[43]	*GJB1*	[43]
TF	[43]	*KRT18*	[44]
KRT8	[45]	*VWF*	[46]
TAT	[47]	*HNF4A*	[48]
TDO2	[48]	*G6PC*	[48]
FOXA2	[49]	*HNF1A*	[49]

brain	Reference	brain	Reference
TH	[50]	*TUBB3*	[50]
GFAP	[50]	*PITX3*	[50]
NR4A2	[50]	*SYT1*	[51]
SYP	[50]	*KCNJ6*	[52]
SLC6A3	[52]	*DBH*	[52]
SLC6A4	[52]	*NES*	[51]
ALDH1A1	[53]	*NCAM1*	[51]
ASCL1	[54]	*HES6*	[54]
DCX	[54]	*DLL3TFF3*	[54]
TFF3	[54]	*PITX2*	[54]
DDC	[54]	*DLL1*	[54]
PINK1	[54]	*SYN1*	[54]
MAP2	[51]	*EN1*	[55]
LMX1A	[55]	*GUCY2C*	[55]
CALB1	[55]	*SLC18A2*	[55]
UNCX	[55]	*PBX1*	[55]
NECAB1	[55]	*C1QL1*	[55]
OTX2	[51]		

[9] Narayanan, K., Schumacher, K.M., Tasnim, F., Kandasamy, K., Schumacher, A., Ni, M., Gao, S., Gopalan, B., Zink, D., Ying, J.Y.: Human embryonic stem cells differentiate into functional renal proximal tubular like cells. Kidney International **83**(4), 593–603 (2013). doi:10.1038/ki.2012.442

[10] Soler, M.J., Wysocki, J., Batlle, D.: ACE2 alterations in kidney disease. Nephrology, dialysis, transplantation : official publication of the European Dialysis and Transplant Association - European Renal Association, 1–10 (2013). doi:10.1093/ndt/gft320

[11] Peran, M., Marchal, J.A., Lopez, E., Jimenez-Navarro, M., Boulaiz, H., Rodriguez-Serrano, F., Carrillo, E., Sanchez-Espin, G., De Teresa, E., Tosh, D., Aranega, A.: Human cardiac tissue induces transdifferentiation of adult stem cells towards cardiomyocytes. Cytotherapy **12**, 332–337 (2010). doi:10.3109/14653240903548202

[12] Chun, J.L., O'Brien, R., Song, M.H., Wondrasch, B.F., Berry, S.E.: Injection of vessel-derived stem cells prevents dilated cardiomyopathy and promotes angiogenesis and endogenous cardiac stem cell proliferation in mdx/utrn-/- but not aged mdx mouse models for duchenne muscular dystrophy. Stem cells translational medicine **2**(1), 68–80 (2013). doi:10.5966/sctm.2012-0107

[13] Zhao, P., Ise, H., Hongo, M., Ota, M., Konishi, I., Nikaido, T.: Human amniotic mesenchymal cells have some characteristics of cardiomyocytes. Transplantation **79**, 528–535 (2005)

[14] Kilic, A., Velic, A., Windt, L.J.D., Fabritz, L., Voss, M., Zwiener, M., Baba, H.A., Eickels, M.V., Schlatter, E.: Enhanced Activity of the Myocardial Na / H Exchanger Natriuretic Peptide Receptor Deficient Mice. Circulation **112**, 2307–17 (2005). doi:10.1161/CIRCULATIONAHA.105.542209

[15] He, Q., Mendez, M., LaPointe, M.C.: Regulation of the human brain natriuretic peptide gene by GATA-4. American Journal of Physiology - Endocrinology And Metabolism **283**, 50–57 (2002)

[16] Jeyaseelan, R., Poizat, C., Baker, R.K., Abdishoo, S., Isterabadi, L.B., Lyons, G.E., Kedes, L.: A novel cardiac-restricted target for doxorubicin. CARP, a nuclear modulator of gene expression in cardiac progenitor cells and cardiomyocytes. The Journal of Biological Chemistry **272**, 22800–22808 (1997)

[17] Rybin, V.O., Grabham, P.W., Elouardighi, H., Steinberg, S.F.: Caveolae-associated proteins in cardiomyocytes: caveolin-2 expression and interactions with caveolin-3. American journal of physiology Heart and circulatory physiology **285**, 325–332 (2003)

[18] Sun, J., Picht, E., Ginsburg, K.S., Bers, D.M., Steenbergen, C., Murphy, E.: Hypercontractile female hearts exhibit increased S-nitrosylation of the

L-type Ca2+ channel alpha1 subunit and reduced ischemia/reperfusion injury. Circulation Research **98**, 403–411 (2006)

[19] Kodama, H., Hirotani, T., Suzuki, Y., Ogawa, S., Yamazaki, K.: Cardiomyogenic Differentiation in Cardiac Myxoma Expressing Lineage-Specific Transcription Factors. The American journal of pathology **161**, 381–389 (2002)

[20] Zang, M.-X., Li, Y., Xue, L.-X., Jia, H.-T., Jing, H.: Cooperative activation of atrial naturetic peptide promoter by dHAND and MEF2C. Journal of Cellular Biochemistry **93**, 1255–1266 (2004)

[21] Bin, Z., Sheng, L.-G., Gang, Z.-C., Hong, J., Jun, C., Bo, Y., Hui, S.: Efficient cardiomyocyte differentiation of embryonic stem cells by bone morphogenetic protein-2 combined with visceral endoderm-like cells. Cell Biology International **30**, 769–76 (2006). doi:10.1016/j.cellbi.2006.05.011

[22] Chiavegato, A., Bollini, S., Pozzobon, M., Callegari, A., Gasparotto, L., Taiani, J., Piccoli, M., Lenzini, E., Gerosa, G., Vendramin, I., Cozzi, E., Angelini, A., Iop, L., Zanon, G.F., Atala, A., De Coppi, P., Sartore, S.: Human amniotic fluid-derived stem cells are rejected after transplantation in the myocardium of normal, ischemic, immuno-suppressed or immuno-deficient rat. Journal of Molecular and Cellular Cardiology **42**, 746–759 (2007)

[23] Chan, K.K., Tsui, S.K., Lee, S.M., Luk, S.C., Liew, C.C., Fung, K.P., Waye, M.M., Lee, C.Y.: Molecular cloning and characterization of FHL2, a novel LIM domain protein preferentially expressed in human heart. Gene **210**, 345–350 (1998)

[24] Vos, M.J., Kanon, B., Kampinga, H.H.: HSPB7 is a SC35 speckle resident small heat shock protein. Biochimica et Biophysica Acta **1793**, 1343–1353 (2009)

[25] Frey, N., Richardson, J.A., Olson, E.N.: Calsarcins, a novel family of sarcomeric calcineurin-binding proteins. Proceedings of the National Academy of Sciences of the United States of America **97**, 14632–14637 (2000)

[26] Ichiyasu, H., Ichikado, K., Yamashita, A., Iyonaga, K., Sakamoto, O., Suga, M., Kohrogi, H.: Pneumocyte biomarkers KL-6 and surfactant protein D reflect the distinct findings of high-resolution computed tomography in non-specific interstitial pneumonia. Respiration; international review of thoracic diseases **83**(3), 190–197 (2012). doi:10.1159/000326924

[27] Ma, N., Gai, H., Mei, J., Ding, F.-B., Bao, C.-R., Nguyen, D.M., Zhong, H.: Bone marrow mesenchymal stem cells can differentiate into type II alveolar epithelial cells in vitro. Cell Biology International **35**, 1261–6 (2011). doi:10.1042/CBI20110026

[28] Salaun, B., de Saint-Vis, B., Pacheco, N., Pacheco, Y., Riesler, A., Isaac, S., Leroux, C., Clair-Moninot, V., Pin, J.-J., Griffith, J., Treilleux, I., Goddard, S., Davoust, J., Kleijmeer, M., Lebecque, S.: CD208/dendritic cell-lysosomal associated membrane protein is a marker of normal and transformed type II pneumocytes. The American journal of pathology **164**(3), 861–871 (2004). doi:10.1016/S0002-9440(10)63174-4

[29] Fujino, N., Kubo, H., Suzuki, T., Ota, C., Hegab, A.E., He, M., Suzuki, S., Suzuki, T., Yamada, M., Kondo, T., Kato, H., Yamaya, M.: Isolation of alveolar epithelial type II progenitor cells from adult human lungs. Laboratory investigation a journal of technical methods and pathology **91**, 363–378 (2011). doi:10.1038/labinvest.2010.187

[30] Foster, C.D., Varghese, L.S., Gonzales, L.W., Margulies, S.S., Guttentag, S.H.: The Rho pathway mediates transition to an alveolar type I cell phenotype during static stretch of alveolar type II cells. Pediatric Research **67**, 585–590 (2010)

[31] Bates, S.R., Tao, J.-Q., Yu, K.J., Borok, Z., Crandall, E.D., Collins, H.L., Rothblat, G.H.: Expression and Biological Activity of ABCA1 in Alveolar Epithelial Cells. American Journal of Respiratory Cell and Molecular Biology **38**, 283–292 (2008). doi:10.1165/rcmb.2007-0020OC

[32] Monzon, M.E., Forteza, R.M., Casalino-matsuda, S.M.: MCP-1 / CCR2B-dependent loop upregulates MUC5AC and MUC5B in human airway epithelium. American Journal of Physiology - Lung Cellular and Molecular Physiology **300**, 204–215 (2011). doi:10.1152/ajplung.00292.2010.

[33] Slager, R.E., Devasure, J.M., Pavlik, J.A., Sisson, J.H., Wyatt, T.A.: RACK1, a PKC Targeting Protein, Is Exclusively Localized to Basal Airway Epithelial Cells. Journal of Histochemistry and Cytochemistry **56**, 7–14 (2008). doi:10.1369/jhc.7A7249.2007

[34] Baraniuk, J.N., Ohkubo, K., Kwon, O.J., Mak, J., Ali, M., Davies, R., Twort, C., Kaliner, M., Letarte, M., Barnes, P.J.: Localization of neutral endopeptidase (NEP) mRNA in human bronchi. The European respiratory journal official journal of the European Society for Clinical Respiratory Physiology **8**, 1458–1464 (1995)

[35] Altraja, S., Jaama, J., Altraja, A.: Proteome changes of human bronchial epithelial cells in response to pro-inflammatory mediator leukotriene E4 and pro-remodelling factor TGF-beta1. Journal of proteomics **73**, 1230–1240 (2010)

[36] Park, S.-A., Lee, Y.-C., Ma, T.-Z., Park, J.-A., Han, M.-K., Lee, H.-H., Kim, H.-G., Kwak, Y.-G.: hKv1.5 channels play a pivotal role in the functions of human alveolar macrophages. Biochemical and Biophysical Research Communications **346**, 567–571 (2006)

[37] Bonfield, T.L., Raychaudhuri, B., Malur, A., Abraham, S., Trapnell, B.C., Kavuru, M.S., Thomassen, M.J.: PU.1 regulation of human alveolar macrophage differentiation requires granulocyte-macrophage colony-stimulating factor. American journal of physiology Lung cellular and molecular physiology **285**, 1132–1136 (2003)

[38] Beaufort, N., Leduc, D., Eguchi, H., Mengele, K., Hellmann, D., Masegi, T., Kamimura, T., Yasuoka, S., Fend, F., Chignard, M., Pidard, D.: The human airway trypsin-like protease modulates the urokinase receptor (uPAR, CD87) structure and functions. American journal of physiology Lung cellular and molecular physiology **292**, 1263–1272 (2007)

[39] Peri, A., Cordella-Miele, E., Miele, L., Mukherjee, A.B.: Tissue-specific expression of the gene coding for human Clara cell 10-kD protein, a phospholipase A2-inhibitory protein. The Journal of clinical investigation **92**(5), 2099–109 (1993). doi:10.1172/JCI116810

[40] Besnard, V., Wert, S.E., Kaestner, K.H., Whitsett, J.A.: Stage-specific regulation of respiratory epithelial cell differentiation by Foxa1. American journal of physiology Lung cellular and molecular physiology **289**, 750–759 (2005)

[41] Bals, R., Welsch, U.: Lectins and antibodies to blood group antigens as markers for the basal cells of the human respiratory epithelium. Microscopy Research and Technique **38**, 505–511 (1997)

[42] Horiguchi, K., Horiguchi, S., Yamashita, N., Irie, K., Masuda, J., Takano-Ohmuro, H., Himi, T., Miyazawa, M., Moriwaki, Y., Okuda, T., Misawa, H., Ozaki, H., Kawashima, K.: Expression of SLURP-1, an endogenous alpha7 nicotinic acetylcholine receptor allosteric ligand, in murine bronchial epithelial cells. Journal of Neuroscience Research **87**, 2740–2747 (2009)

[43] Aurich, H., Sgodda, M., Kaltwasser, P., Vetter, M., Weise, a., Liehr, T., Brulport, M., Hengstler, J.G., Dollinger, M.M., Fleig, W.E., Christ, B.: Hepatocyte differentiation of mesenchymal stem cells from human adipose tissue in vitro promotes hepatic integration in vivo. Gut **58**(4), 570–581 (2009). doi:10.1136/gut.2008.154880

[44] Weiss, T.S., Lichtenauer, M., Kirchner, S., Stock, P., Aurich, H., Christ, B., Brockhoff, G., Kunz-Schughart, L.A., Jauch, K.-W., Schlitt, H.-J., Thasler, W.E.: Hepatic progenitor cells from adult human livers for cell transplantation. Gut **57**, 1129–1138 (2008)

[45] Sana, G., Lombard, C., Vosters, O., Jazouli, N., Andre, F., Stephenne, X., Smets, F., Najimi, M., Sokal, E.M.: Adult human hepatocytes promote CD4+ T cell hyporesponsiveness via interleukin-10 producing allogeneic dendritic cells. Cell transplantation, 1–38 (2013). doi:10.3727/096368913X666421

[46] Gerlach, J.C., Over, P., Turner, M.E., Thompson, R.L., Foka, H.G., Chen, W.C.W., Péault, B., Gridelli, B., Schmelzer, E.: Perivascular mesenchymal progenitors in human fetal and adult liver. Stem cells and development **21**(18), 3258–3269 (2012). doi:10.1089/scd.2012.0296

[47] Liang, X.J., Chen, X.J., Yang, D.H., Huang, S.M., Sun, G.D., Chen, Y.P.: Differentiation of human umbilical cord mesenchymal stem cells into hepatocyte-like cells by hTERT gene transfection in vitro. Cell Biol Int **36**, 215–221 (2012)

[48] Maerckx, C., Scheers, I., Tondreau, T., Campard, D., Nyabi, O., Najimi, M., Sokal, E.: Hepato-biliary profile of potential candidate liver progenitor cells from healthy rat liver. World journal of gastroenterology WJG **18**, 3511–9 (2012). doi:10.3748/wjg.v18.i27.3511

[49] Cho, J.-w., Lee, C.-y., Ko, Y.: Therapeutic potential of mesenchymal stem cells overexpressing human forkhead box A2 gene in the regeneration of damaged liver tissues. Journal of Gastroenterology and Hepatology **27**, 1362–1370 (2012). doi:10.1111/j.1440-1746.2012.07137.x

[50] Daadi, M.M., Grueter, B.a., Malenka, R.C., Redmond, D.E., Steinberg, G.K.: Dopaminergic neurons from midbrain-specified human embryonic stem cell-derived neural stem cells engrafted in a monkey model of Parkinson's disease. PloS one **7**(7), 41120 (2012). doi:10.1371/journal.pone.0041120

[51] Pruszak, J., Sonntag, K.-C., Aung, M.H., Sanchez-Pernaute, R., Isacson, O.: Markers and methods for cell sorting of human embryonic stem cell-derived neural cell populations. Stem Cells **25**, 2257–2268 (2007)

[52] Sanchez-Danes, A., Consiglio, A., Richaud, Y., Rodriguez-Piza, I., Dehay, B., Edel, M., Bove, J., Memo, M., Vila, M., Raya, A., Izpisua Belmonte, J.C.: EFFICIENT GENERATION OF A9 MIDBRAIN DOPAMINERGIC NEURONS BY LENTIVIRAL DELIVERY OF LMX1A IN HUMAN EMBRYONIC STEM CELLS AND iPS CELLS. Human Gene Therapy **23**, 56–69 (2011). doi:10.1089/hum.2011.054

[53] Decressac, M., Mattsson, B., Weikop, P., Lundblad, M., Jakobsson, J., Björklund, A.: TFEB-mediated autophagy rescues midbrain dopamine neurons from α-synuclein toxicity. Proceedings of the National Academy of Sciences of the United States of America (2013). doi:10.1073/pnas.1305623110

[54] Kriks, S., Shim, J.-W., Piao, J., Ganat, Y.M., Wakeman, D.R., Xie, Z., Carrillo-Reid, L., Auyeung, G., Antonacci, C., Buch, A., Yang, L., Beal, M.F., Surmeier, D.J., Kordower, J.H., Tabar, V., Studer, L.: Dopamine neurons derived from human ES cells efficiently engraft in animal models of Parkinsons disease. Nature **480**, 0–6 (2011). doi:10.1038/nature10648

[55] Ganat, Y.M., Calder, E.L., Kriks, S., Nelander, J., Tu, E.Y., Jia, F., Battista, D., Harrison, N., Parmar, M., Tomishima, M.J., Rutishauser, U., Studer, L.: Identification of embryonic stem cell-derived midbrain dopaminergic neurons for engraftment. J Clin Invest **122**, 2928–2939 (2012)
