# Supplementary material for: MGFM: a novel tool for detection of tissue and cell specific marker genes from microarray gene expression data
Source: BMC Genomics. 2015 Aug 28;16(1):645. doi: 10.1186/s12864-015-1785-9 (PMC4552366; doi:10.1186/s12864-015-1785-9)
Supplement: Additional file 2 — Plots of Precision/Recall comparing our method to t -test. This file includes Plots of Precision/Recall comparing MGFM to t-test. (462KB PDF) [file 12864_2015_1785_MOESM2_ESM.pdf]

Plots of Precision/Recall comparing our method to *t*-test

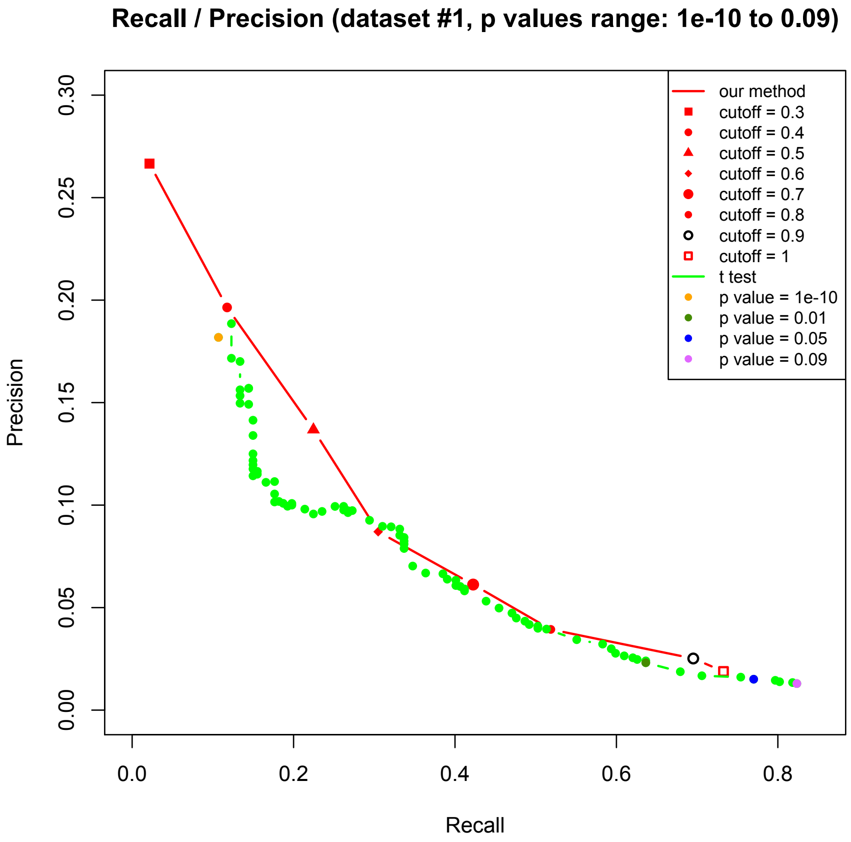

Figure 1: Comparison of Precision/Recall for the complete set of genes selected by our tool and *t*-test as potential markers for the examined tissues using data set #1.

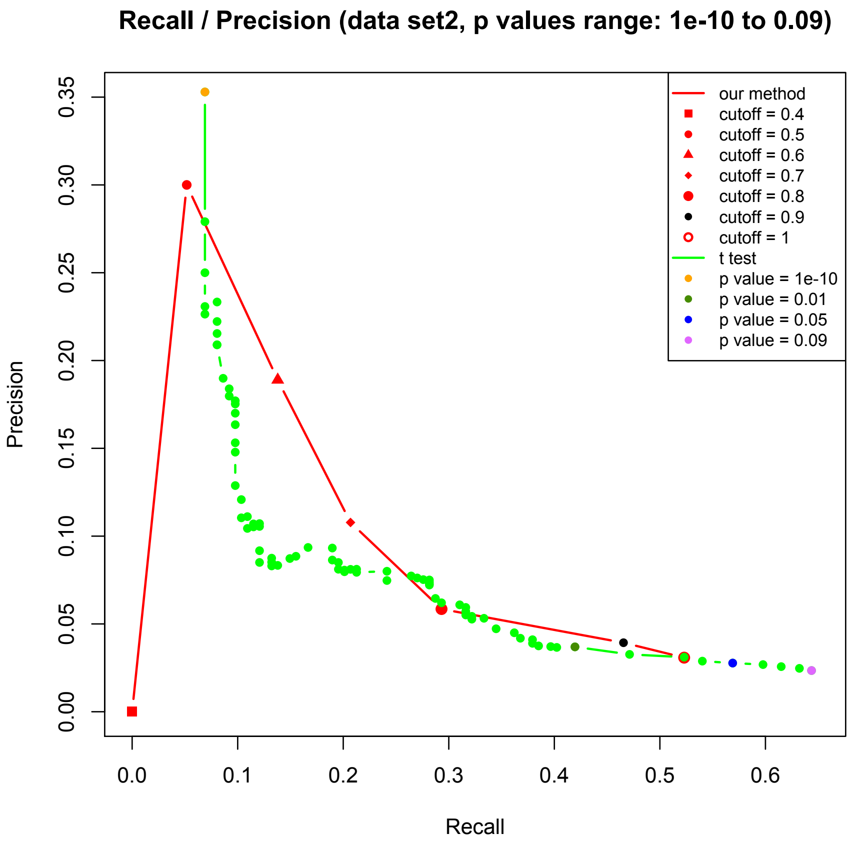

Figure 2: Comparison of Precision/Recall for the complete set of genes selected by our tool and *t*-test as potential markers for the examined tissues using data set #2.
