# Supplementary material for: MGFM: a novel tool for detection of tissue and cell specific marker genes from microarray gene expression data
Source: BMC Genomics. 2015 Aug 28;16(1):645. doi: 10.1186/s12864-015-1785-9 (PMC4552366; doi:10.1186/s12864-015-1785-9)
Supplement: Additional file 3 — Gel electrophoresis images. This file includes the gel electrophoresis images (Figures S1–S11). (981KB PDF) [file 12864_2015_1785_MOESM3_ESM.pdf]

Gel electrophoresis images

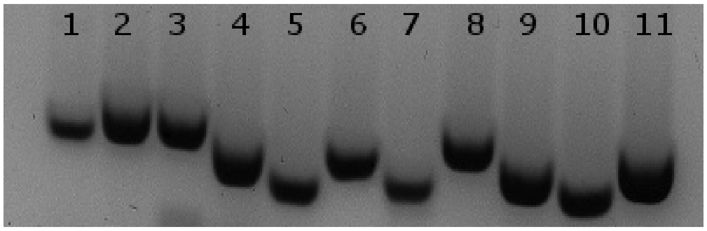

Liver

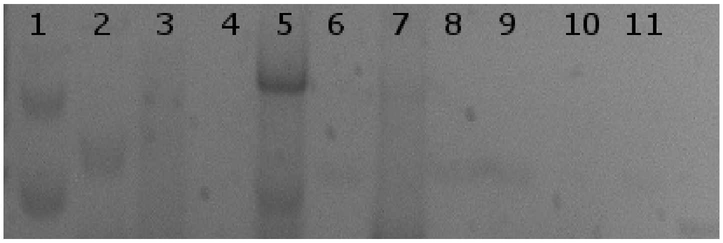

Lung

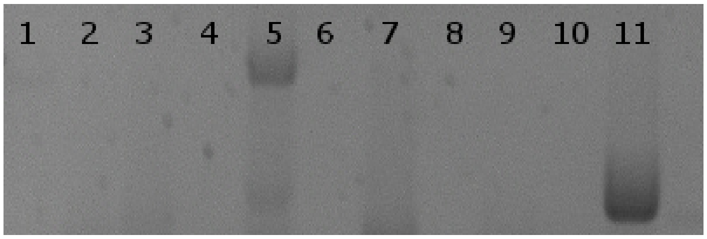

Heart

Figure 1: Expression of human gene transcripts predicted by our tool as markers for liver, detected by PCR reaction and agarose gel electrophoresis in liver, lung and heart.
1: *AKR1D1*, 2: *APOA2*, 3: *CYP2C8*, 4: *GC*, 5: *CPS1*, 6: *CYP2E1*, 7: *SERPINC1*, 8: *AHSG*, 9: *APOC3*, 10: *AMBP*, 11: *FGG*

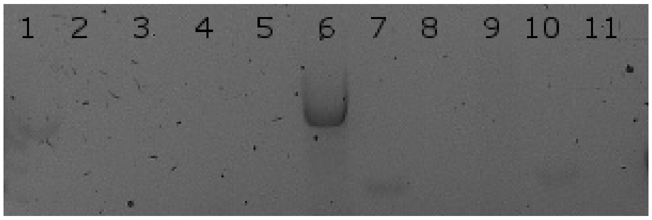

Brain

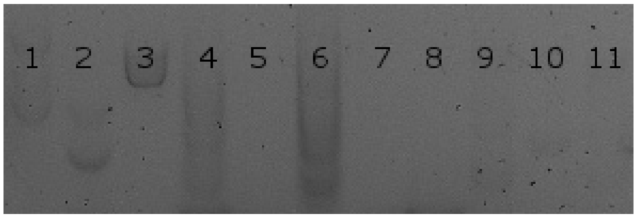

Kidney

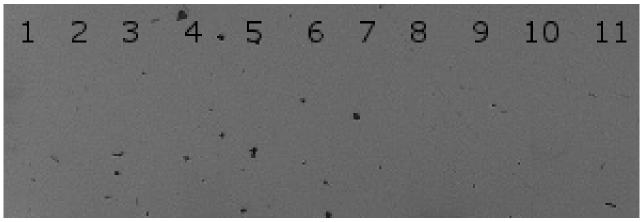

Negative Control

Figure 2: Expression of human gene transcripts predicted by our tool as markers for liver, detected by PCR reaction and agarose gel electrophoresis in brain, kidney and the negative control.
1: *AKR1D1*, 2: *FGG*, 3: *APOA2* 4: *CYP2C8*, 5: *GC*, 6: *CPS1*, 7: *CYP2E1*, 8: *APOC3*, 9: *SERPINC1*, 10: *AHSG*, 11: *AMBP*

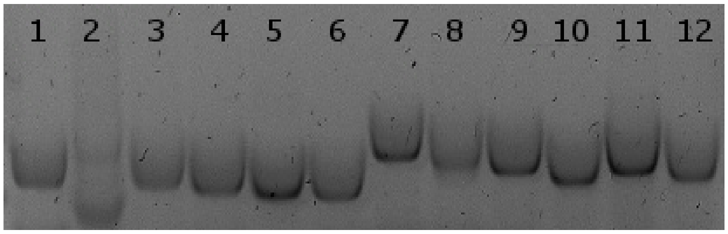

Lung

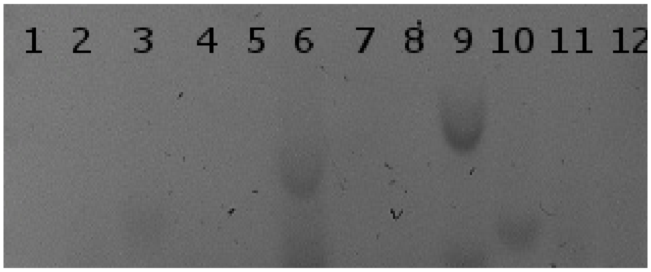

Liver

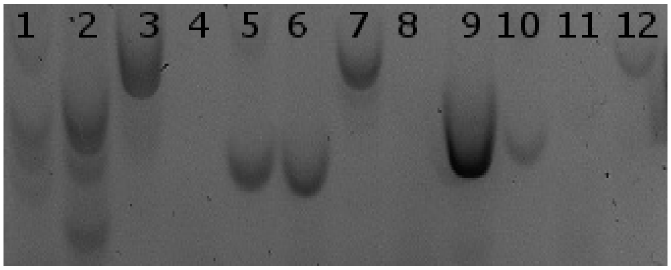

Heart

Figure 3: Expression of human gene transcripts predicted by our tool as markers for lung, detected by PCR reaction and agarose gel electrophoresis in lung, liver and heart.
1: *CLDN18*, 2: *NKX2-1*, 3: *SCGB1A1*, 4: *SFTPB*, 5: *CYP4B1*, 6: *CD52*, 7: *LAMP3*, 8: *AGER*, 9: *LYZ*, 10: *SFTPD*, 11: *SFTPC*, 12: *SLC34A2*

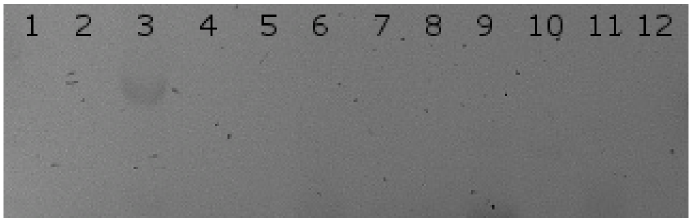

Brain

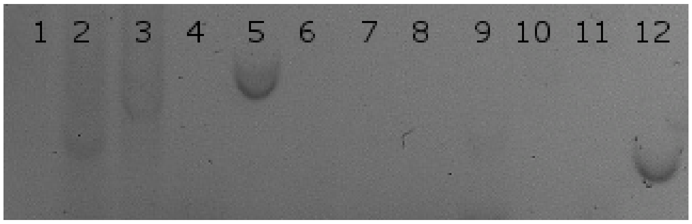

Kidney

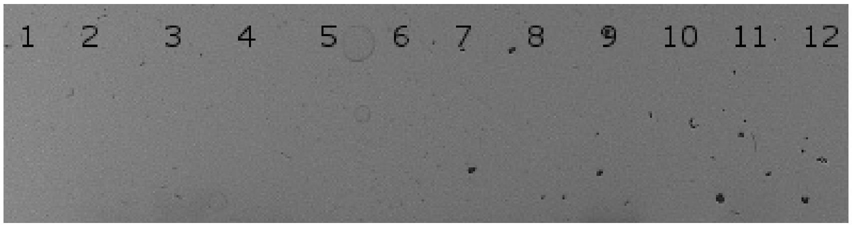

Negative Control

Figure 4: Expression of human gene transcripts predicted by our tool as markers for lung, detected by PCR reaction and agarose gel electrophoresis in brain, kidney and the negative control.
1: *CLDN18*, 2: *NKX2-1*, 3: *SCGB1A1*, 4: *SFTPB*, 5: *CYP4B1*, 6: *CD52*, 7: *LAMP3*, 8: *AGER*, 9: *LYZ*, 10: *SFTPD*, 11: *SFTPC*, 12: *SLC34A2*

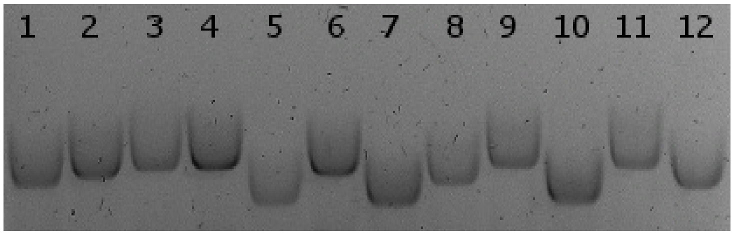

Heart

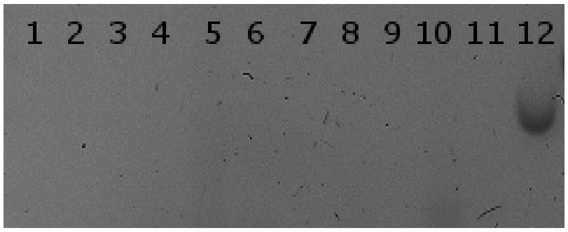

Liver

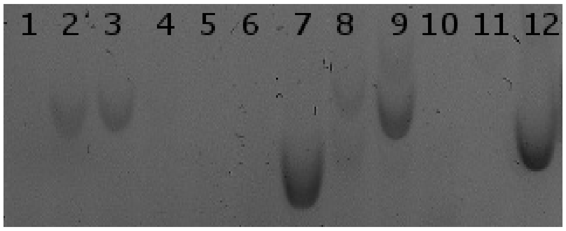

Lung

Figure 5: Expression of human gene transcripts predicted by our tool as markers for heart, detected by PCR reaction and agarose gel electrophoresis in heart, liver and lung.
1: *MYOZ2*, 2: *TNNI3*, 3: *SYNPO2L*, 4: *MYH6*, 5: *CSRP3*, 6: *CKM*, 7: *PLN*, 8: *MB*, 9: *TTN*, 10: *MYL7*, 11: *MYH7*, 12: *TPM1*

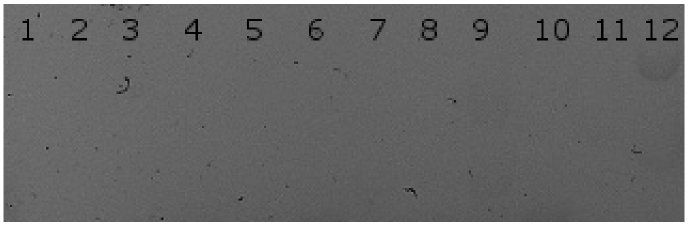

Brain

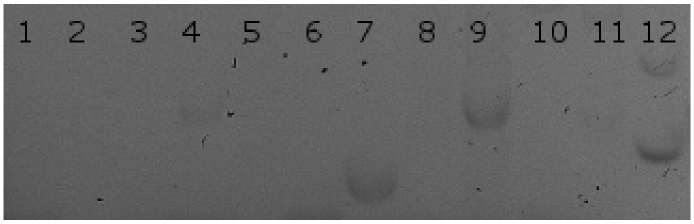

Kidney

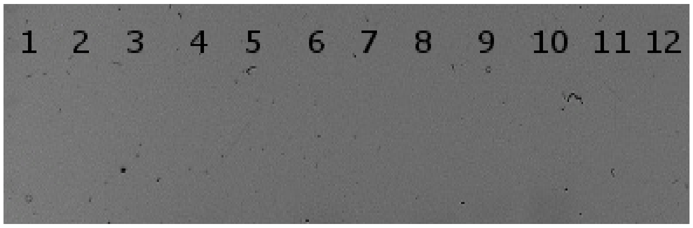

Negative control

Figure 6: Expression of human gene transcripts predicted by our tool as markers for heart, detected by PCR reaction and agarose gel electrophoresis in brain, kidney and the negative control. 1: *MYOZ2*, 2: *TNNI3*, 3: *SYNPO2L*, 4: *MYH6*, 5: *CSRP3*, 6: *CKM*, 7: *PLN*, 8: *MB*, 9: *TTN*, 10: *MYL7*, 11: *MYH7*, 12: *TPM1*

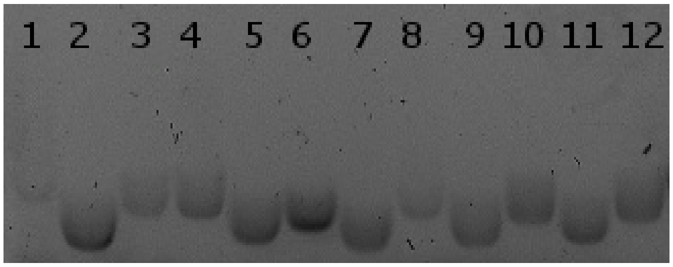

Brain

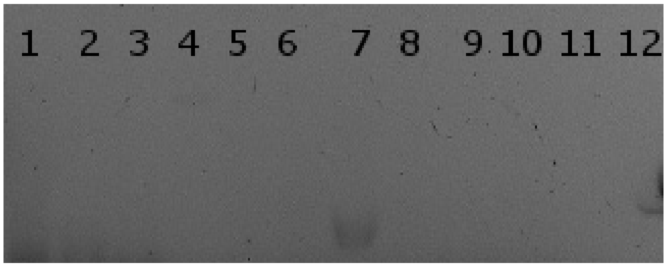

Liver

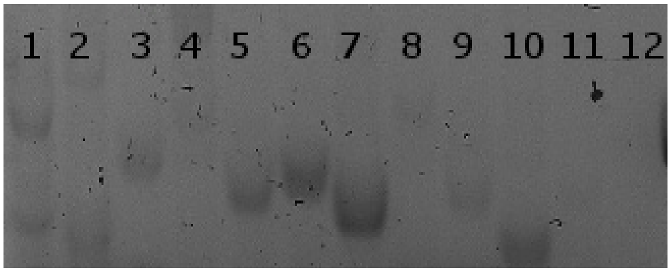

Lung

Figure 7: Expression of human gene transcripts predicted by our tool as markers for brain, detected by PCR reaction and agarose gel electrophoresis in brain, liver and lung.
1: *GAP43*, 2: *GFAP*, 3: *TMEFF1*, 4: *FUT9*, 5: *SYT1*, 6: *SNAP25*, 7: *MBP*, 8: *GRIA2*, 9: *KIFSC*, 10: *STMN2*, 11: *NEFM*, 12: *GABBR2*

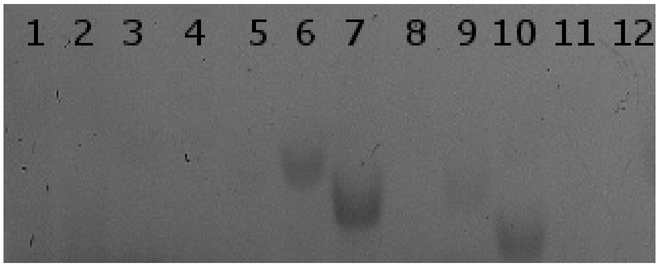

Heart

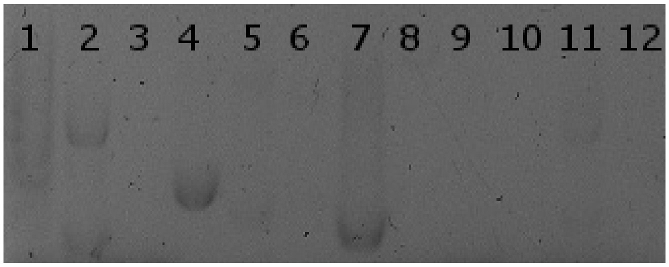

Kidney

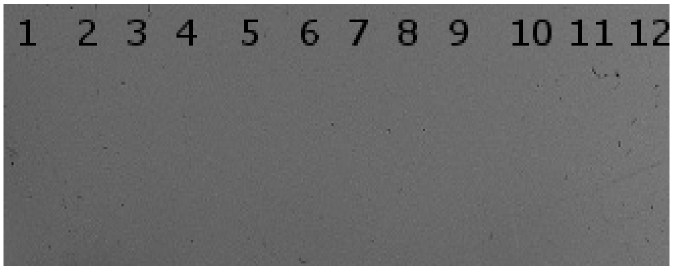

Negative control

Figure 8: Expression of human gene transcripts predicted by our tool as markers for brain, detected by PCR reaction and agarose gel electrophoresis in heart, kidney and the negative control. 1: *GAP43*, 2: *GFAP*, 3: *TMEFF1*, 4: *FUT9*, 5: *SYT1*, 6: *SNAP25*, 7: *MBP*, 8: *GRIA2*, 9: *KIFSC*, 10: *STMN2*, 11: *NEFM*, 12: *GABBR2*

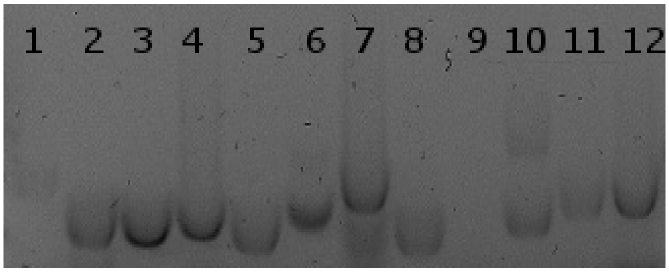

Kidney

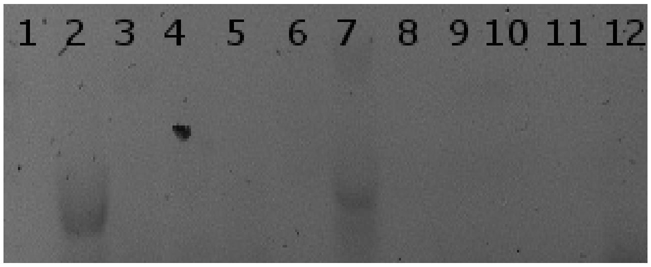

Liver

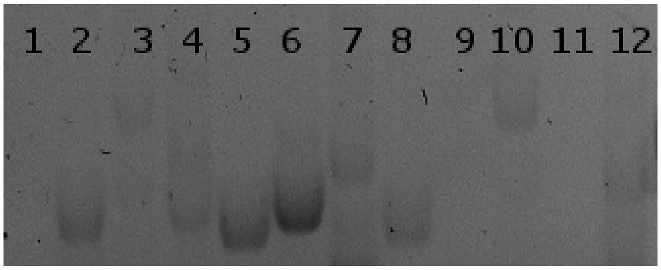

Lung

Figure 9: Expression of human gene transcripts predicted by our tool as markers for kidney, detected by PCR reaction and agarose gel electrophoresis in kidney, liver and lung.
1: *SLC12A1*, 2: *SLC3A1*, 3: *UMOD*, 4: *AOC1*, 5: *CD24*, 6: *HSD11B2*, 7: *CA12*, 8: *PDZK1IP1*, 9: *FXYD2*, 10: *CDH16*, 11: *SLC22A8*, 12: *CLDN8*

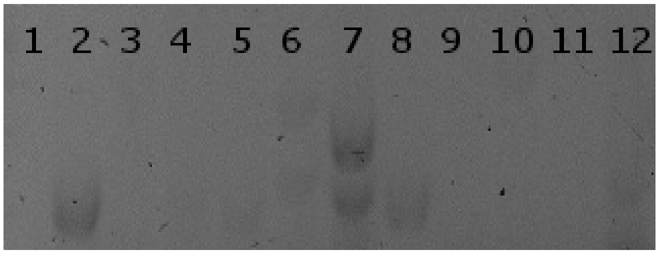

Heart

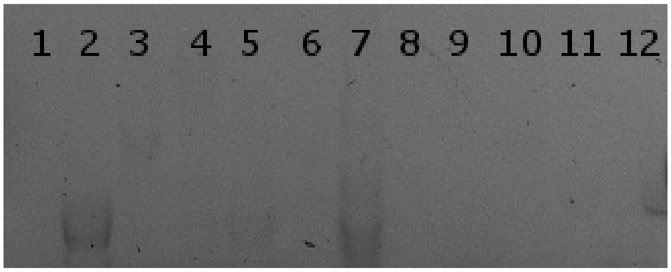

Brain

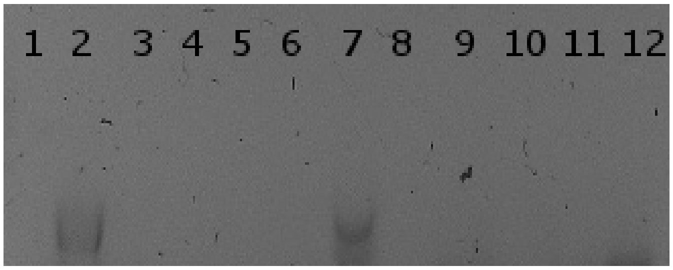

Negative control

Figure 10: Expression of human gene transcripts predicted by our tool as markers for kidney, detected by PCR reaction and agarose gel electrophoresis in heart, brain and the negative control.
1: *SLC12A1*, 2: *SLC3A1*, 3: *UMOD*, 4: *AOC1*, 5: *CD24*, 6: *HSD11B2*, 7: *CA12*, 8: *PDZK1IP1*, 9: *FXYD2*, 10: *CDH16*, 11: *SLC22A8*, 12: *CLDN8*

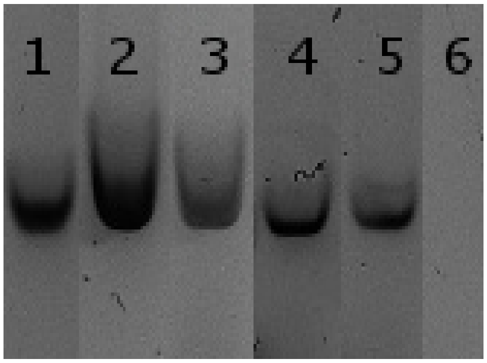

Figure 11: Expression of the human gene transcript β-actin, detected by PCR reaction and agarose gel electrophoresis in liver (1), lung (2), heart (3), brain (4), kidney (5) and the negative control (6).
