# Supplementary material for: MGFM: a novel tool for detection of tissue and cell specific marker genes from microarray gene expression data
Source: BMC Genomics. 2015 Aug 28;16(1):645. doi: 10.1186/s12864-015-1785-9 (PMC4552366; doi:10.1186/s12864-015-1785-9)
Supplement: Additional file 4 — Description of the predicted marker genes. (126KB PDF) [file 12864_2015_1785_MOESM4_ESM.pdf]

Description of the predicted marker genes

Liver marker genes

The 11 liver marker genes that we identified and validated with RT-PCR are *AKR1D1*, *FGG*, *APOA2*, *CYP2C8*, *GC*, *CPS1*, *CYP2E1*, *APOC3*, *SERPINC1*, *AHSG*, and *AMBP*. We confirmed by PCR the liver-specific expression of *CYP2C8*, *GC*, *CPS1*, *CYP2E1*, *APOC3*, *SERPINC1*, *AHSG*, and *AMBP*.
AMBP gene encodes the two plasma glycoproteins alpha-1-Microglobulin (A1M) and bikunin. *A1M* belongs to the superfamily of lipocalin transport proteins and may play a role in the regulation of inflammatory processes, whereas bikunin is an urinary trypsin inhibitor. The strict liver-specific transcription of the *AMBP* gene appears to be due to the presence of a strong liver-specific enhancer [1]. *AHSG* is synthesized by hepatocytes and secreted into serum. It is involved in insulin resistance and fat accumulation in the liver [2]. *CYP2C8* and *CYP2E1* encode members of the cytochrome P450 superfamily of enzymes. Cytochrome P450 epoxygenases are predominantly expressed in the liver [3] and they catalyze many reactions involved in drug metabolism and synthesis of cholesterol, steroids and other lipids. *GC* encodes a protein that belongs to the albumin gene family. It functions predominantly as a transporter protein for vitamin D and its metabolites [4]. *APOC3* is a very low density lipoprotein (VLDL) protein. *APOC3* inhibits lipoprotein lipase and lipoprotein remnant uptake by the liver [5, 6]. *SERPINC1* is a plasma protease inhibitor, synthesized in the liver and a member of the serpin superfamily. It is the principal plasma serpin of blood coagulation proteases and functions as inhibitor of thrombin and other factors by the formation of covalently linked complexes.

Lung marker genes

Lung marker genes identified in the current study include *CLDN18*, *NKX2-1*, *SCGB1A1*, *SFTPB*, *CYP4B1*, *CD52*, *LAMP3*, *AGER*, *LYZ*, *SFTPD*, *SFTPC*, and *SLC34A2*. Among them, genes that have been described or used as markers of lung such as *SGB1A1* [7], *LAMP3* [8], *SFTPC* [9], and *SFTPD* [10]. Genes uniquely expressed in the lung (compared with the other 4 tissues) confirmed by PCR include *CLDN18*, *SFTPB*, *SFTPC*, *SFTPD* and *AGER*. In addition, the set of predicted marker genes comprised genes of particular importance in lung, that were also detected in heart, such as *NKX2-1*, *SCGB1A1*, *CYP4B1*, and *LAMP3*.
CLDNs are components of cellular tight junctions regulating the permeability of cellular layers between different tissue compartments [11]. Mutations in *CLDN18* are associated with adenocarcinomas [12]. *NKX2-1* is a homeodomain-containing transcription factor, encoding thyroid transcription factor-1 (*TTF-1*). *NKX2-1* plays a crucial role in normal lung function and morphogenesis [13], and has been suggested as a lineage marker for the terminal respiratory unit in lung carcinogenesis [14]. *SCGB1A1* is a member of the secretoglobin family of small secreted proteins, implicated in numerous functions including

anti-inflammation. Defects in *SCGB1A1* are associated with a susceptibility to asthma. *SFTPB*, *SFTPC*, and *SFTPD* are surfactant proteins which are involved in defense against microbial invasion. Moreover, they act by lowering the surface tension and are crucial for gaseous exchange between air and blood. *LAMP3* is the third member of the lysosome-associated membrane glycoprotein (*LAMP*) family, and it has been reported to play important roles in the occurrence and metastasis of lung cancer [15]. *AGER*, also known as *RAGE*, is a cell surface receptor. *AGER* is highly expressed in the lung, in particular alveolar epithelial cells [16]. *AGER* expression is significantly decreased in human lung carcinomas [17], which suggests that *AGER* may function in suppressing lung cancer. *SLC34A2* is a phosphate transport protein. Mutations in the corresponding gene has been associated with pulmonary alveolar microlithiasis [18]. *LYZ* is an essential component of innate defense in lung epithelia.
Lindskog et al. [19] analyzed the lung-specific transcriptome in comparison to 26 other tissue types based on RNA-Seq combined with antibody-based protein profiling, and reported 20 genes (*SFTPA1*, *SFTPB*, *SFTPC*, *SFTPA2*, *SCGB3A2*, *AGER*, *SFTPD*, *ROS1*, *SCGB1A1*, *MS4A15*, *RTKN2*, *SLC34A2*, *NAPSA*, *SFTA2*, *LRRN4*, *LAMP3*, *CACNA2D2*, *FAM92B*, *CCDC17*, and *LDLRAD1*) with higher expression levels in the lung as compared to all the other 26 analyzed tissues. Among these 20 genes 15 genes (*SFTPB*, *SFTPC*, *SFTPA2*, *SCGB3A2*, *AGER*, *SFTPD*, *SCGB1A1*, *MS4A15*, *RTKN2*, *SLC34A2*, *NAPSA*, *SFTA2*, *LRRN4*, *LAMP3*, and *CACNA2D2*) were estimated by our tool as lung markers using data set 1 or 2. From the remaining 5 genes 3 genes (*SFTPA1*, *FAM92B*, and *LDLRAD1*) were not found on the microarrays of the used data sets. Moreover, from the 12 estimated lung specific genes tested here with PCR, Lindskog et al. [19] reported 7 genes (*SFTPB*, *SFTPC*, *SFTPD*, *SLC34A2*, *LAMP3*, *AGER*, and *NKX2-1*) as significantly associated with survival in lung adenocarcinoma.

Heart marker genes

Using our approach the following genes were highly expressed in the heart compared to the other tissues: *MYOZ2*, *TNNI3*, *SYNPO2L*, *MYH6*, *CSRP3*, *CKM*, *PLN*, *MB*, *TTN*, *MYL7*, *MYH7*, and *TPM1*. We confirmed by PCR the heart-specific expression of *MYOZ2*, *MYH6*, *CSRP3*, *CKM*, *MB*, *MYL7*, and *MYH7*. The remaining genes *TNNI3*, *SYNPO2L*, *PLN*, *TTN*, and *TPM1* that were detected additionally in lung or kidney, were reported to play important roles in the heart function.
MYOZ2 belongs to a family of sarcomeric proteins that bind to calcineurin. Mutations in this gene cause cardiomyopathy familial hypertrophic type 16, a hereditary heart disorder. *MYH6* encodes the alpha heavy chain subunit of cardiac myosin. *CSRP3* plays an important role in the organization of cytosolic structures in cardiomyocytes. Mutations in this gene has been associated with hypertrophic cardiomyopathy and dilated cardiomyopathy [20]. *CKM* is a cytoplasmic enzyme involved in energy homeostasis and is an important serum marker for myocardial infarction. *PLN* has been postulated to regulate the

activity of the calcium pump of cardiac sarcoplasmic reticulum. *MYL7* binds calcium and has been shown to be a useful molecular marker for cardiac chamber specification. *MYH7* encodes the beta heavy chain subunit of cardiac myosin. Mutations in this gene are associated with familial hypertrophic cardiomyopathy, myosin storage myopathy, and dilated cardiomyopathy. *TNNI3* is one of 3 subunits that form the troponin complex of the thin filaments of striated muscle. It serves as a calcium-sensitive switch that regulates striated muscle contraction [21]. Mutations in this gene have been associated with familial hypertrophic cardiomyopathy type 7 and familial restrictive cardiomyopathy. *MB* functions as an intracellular oxygen carrier in the skeletal and heart muscles of most vertebrates [22]. *SYNPO2L* encodes a cytoskeletal protein. Beqqali et al. [23] recently reported the corresponding protein as a novel protein that interacts and colocalizes with α-actinin at the Z-disc of the sarcomere. *TTN* encodes a large abundant protein of striated muscle. Mutations in this gene have been identified in patients with peripartum cardiomyopathy and dilated cardiomyopathy [24]. *TPM1* is a member of the tropomyosin family of highly conserved actin binding proteins, which are involved in the control of thin filament function in striated muscle contraction. Mutations in this gene are associated with type 3 familial hypertrophic cardiomyopathy.

Brain marker genes

The 12 brain marker genes that we identified and verified with RT-PCR are *GAP43*, *GFAP*, *TMEFF1*, *FUT9*, *SYT1*, *SNAP25*, *MBP*, *GRIA2*, *KIF5C*, *STMN2*, *NEFM*, and *GABBR2*. We confirmed by PCR the brain-specific expression of *GFAP*, *TMEFF1*, *SYT1*, *GRIA2*, *KIF5C*, *STMN2*, *NEFM*, and *GABBR2*. *FUT9* was additionally detected in kidney, *SNAP25* in lung and heart, whereas *GAP43* was not detected in any of the examined tissues.

GFAP encodes one of a family of intermediate filament proteins. *GFAP* is known as astrocytes marker. Mutations in this gene have been associated with Alexander disease [25], a rare disorder of astrocytes in the central nervous system. *TMEFF1* encodes a transmembrane protein containing two follistatin-like modules and an epidermal growth factor-like domain. Gery et al. [26] investigated *TMEFF1* expression in normal brain and brain cancer cells. According to this study, *TMEFF1* was highly expressed in the normal brain and at lower levels in brain cancer. These results suggest that *TMEFF1* may function as a tumor suppressor gene in brain cancers. *KIF5C* gene encodes a member of the kinesin superfamily of proteins that are motor proteins involved in various processes in the brain, such as neuronal functioning, development, and survival [27, 28]. In a recent study, Willemsen et al. [28] suggested that mutations in *KIF4A* and *KIF5C* cause intellectual disability by tipping the balance between excitatory and inhibitory synaptic excitability. *STMN2*, also known as *SCG10*, encodes a member of the stathmin family of phosphoproteins, which are involved in microtubule dynamics and signal transduction. Reductions in the expression of this gene have been associated with Down's syndrome[29] and Alzheimer's disease [30] *GRIA2* encodes the *GLUR2* subunit of α-amino-3-

hydroxy-5-methyl-4-isoxazolepropionic acid receptors, which is mainly expressed in the brain, and play important roles in normal brain function [31]. In addition, Hackmann et al. [32] hypothesized that *GRIA2* is involved in intellectual disability. *GABBR2* encodes a membrane protein from the G-protein coupled receptor 3 family and Gamma-aminobutyric acid type B (*GABA-B*) receptor subfamily. The *GABA-B* receptors inhibit neuronal activity through G protein-coupled second-messenger systems, which regulate the release of neurotransmitters and the activity of ion channels and adenylyl cyclase [33, 34]. *GABBR2* have been associated with autism [35]. Additionally, significant reductions in *GABBR1* and *GABBR2* expression in lateral cerebellum of subjects with schizophrenia, bipolar disorder, and major depression have been reported [36]. *NEFM* encodes the medium neurofilament protein, one of the 3 subunits forming the neurofilaments, that localizes to neuronal axons and dendrites [37]. Various human brain diseases have been associated with neurofilaments proteins. *SYT1* encodes an integral membrane protein of synaptic vesicles, which is thought to serve as Ca(2+) sensors in the process of vesicular trafficking and exocytosis. Calcium binding to synaptotagmin-1 participates in triggering neurotransmitter release at the synapse [38]. The corresponding protein was suggested to be important for synaptic function and may be related to cognitive impairments in Alzheimer's disease [39]. *SNAP25* encodes a presynaptic plasma membrane protein involved in the regulation of neurotransmitter release. *SNAP25* is expressed by neurons in the hippocampus, is suggested to play major role in long-term memory formation and has been associated with cognitive ability [40, 41]. *FUT9* encodes a member of the glycosyltransferase family, and has been reported to synthesize the Lewis X carbohydrate structure in the brain [42].

Kidney marker genes

The list of genes selectively expressed in the kidney comprised *SLC12A1*, *SLC3A1*, *UMOD*, *AOC1*, *CD24*, *HSD11B2*, *CA12*, *PDZK1IP1*, *FXYD2*, *CDH16*, *SLC22A8*, and *CLDN8*. Our PCR data confirmed the selective expression of *UMOD*, *AOC1*, *PDZK1IP1*, *CDH16*, *SLC22A8*, and *CLDN8* in kidney. *CD24* and *HSD11B2* were expressed additionally in lung, whereas four genes namely *SLC12A1*, *SLC3A1*, *CA12*, and *FXYD2* were not detected. In contrast to the other tissue specimens, the kidney sample was frozen and stored since two years. This may negatively have affected the RNA stability. Hence, this may explain the absence of four of the predicted markers of kidney.

UMOD encodes a glycoprotein, also known as Tamm-Horsfall potein, which is produced by renal cells of ascending limb of loop of Henle and is largely excreted in urine [43]. *FXYD2* encodes a member of the *FXYD* family of transmembrane proteins, which regulates the function of the Na,K-ATPase in mammalian kidney epithelial cells [44, 45]. *CDH16* encodes Kidney-specific (Ksp)-cadherin, a member of the cadherin superfamily of calcium-dependent cell adhesion molecules. It has been identified as a specific marker for terminal differentiation of the basolateral membrane of renal tubular epithelial cells [46, 47]. Additionally,

Ksp-cadherin has been identified as a highly sensitive marker for chromophobe renal cell carcinoma and oncocytoma [48]. *PDZK1IP1*, also known as *MAP17*, encodes a membrane associated protein. *PDZK1IP1* was earlier detected in normal renal proximal tubules [49]. In addition, it has been reported to be up-regulated in carcinomas arising from kidney, colon, lung, and breast [50]. *AOC1* (formerly known as amiloride-binding protein 1) is a homodimeric glycoprotein, which deaminates putrescine and histamine. *AOC1* was described to be highly expressed in the kidney [51], placenta [52] and intestine [53]. *CLDN8* encodes a member of the claudin family. Claudins are integral membrane proteins of the tight junction that are involved in the permeation of solutes across epithelia via the paracellular pathway. Yu et al. [54] suggested that *CLDN8* plays an important role in the paracellular cation barrier of the distal renal tubule. *SLC22A8* is a member of the organic anion transporter *SLC22* gene family. Cha et al. [55] reported that *SLC22A8* is exclusively expressed in the kidney, and plays important roles in the basolateral uptake of organic anions in proximal tubular cells. *HSD11B2* encodes the type II isoform of 11-beta-hydroxysteroid dehydrogenase, a microsomal enzyme complex responsible for the interconversion of biologically active cortisol and inactive cortisone. *HSD11B2* is highly expressed in kidney and placenta [56].

References

[1] P, R., G, R., P, R., J, S.: An array of binding sites for hepatocyte nuclear factor 4 of high and low affinities modulates the liver-specific enhancer for the human α1-microglobulin/bikunin precursor. Biochem. J. **334**, 577–584 (1998)

[2] Stefan, N.: 2-Heremans-Schmid Glycoprotein/ Fetuin-A Is Associated With Insulin Resistance and Fat Accumulation in the Liver in Humans. Diabetes Care **29**(4), 853–857 (2006). doi:10.2337/diacare.29.04.06.dc05-1938

[3] Zanger, U.M., Schwab, M.: Cytochrome P450 enzymes in drug metabolism: regulation of gene expression, enzyme activities, and impact of genetic variation. Pharmacology & therapeutics **138**(1), 103–41 (2013). doi:10.1016/j.pharmthera.2012.12.007

[4] Cooke, N.E., David, E.V.: Serum vitamin D-binding protein is a third member of the albumin and alpha fetoprotein gene family. The Journal of clinical investigation **76**(6), 2420–4 (1985). doi:10.1172/JCI112256

[5] Virgil Brown, W., Baginsky, M.L.: Inhibition of lipoprotein lipase by an apoprotein of human very low density lipoprotein. Biochemical and Biophysical Research Communications **46**(2), 375–382 (1972). doi:10.1016/S0006-291X(72)80149-9

[6] Windler, E., Havel, R.: Inhibitory effects of C apolipoproteins from rats and humans on the uptake of triglyceride-rich lipoproteins and their remnants by the perfused rat liver. J. Lipid Res. **26**(5), 556–565 (1985)

[7] Peri, A., Cordella-Miele, E., Miele, L., Mukherjee, A.B.: Tissue-specific expression of the gene coding for human Clara cell 10-kD protein, a phospholipase A2-inhibitory protein. The Journal of clinical investigation **92**(5), 2099–109 (1993). doi:10.1172/JCI116810

[8] Salaun, B., de Saint-Vis, B., Pacheco, N., Pacheco, Y., Riesler, A., Isaac, S., Leroux, C., Clair-Moninot, V., Pin, J.-J., Griffith, J., Treilleux, I., Goddard, S., Davoust, J., Kleijmeer, M., Lebecque, S.: CD208/dendritic cell-lysosomal associated membrane protein is a marker of normal and transformed type II pneumocytes. The American journal of pathology **164**(3), 861–871 (2004). doi:10.1016/S0002-9440(10)63174-4

[9] Ma, N., Gai, H., Mei, J., Ding, F.-B., Bao, C.-R., Nguyen, D.M., Zhong, H.: Bone marrow mesenchymal stem cells can differentiate into type II alveolar epithelial cells in vitro. Cell Biology International **35**, 1261–6 (2011). doi:10.1042/CBI20110026

[10] Ichiyasu, H., Ichikado, K., Yamashita, A., Iyonaga, K., Sakamoto, O., Suga, M., Kohrogi, H.: Pneumocyte biomarkers KL-6 and surfactant protein D reflect the distinct findings of high-resolution computed tomography in non-specific interstitial pneumonia. Respiration; international review of thoracic diseases **83**(3), 190–197 (2012). doi:10.1159/000326924

[11] Soini, Y.: Expression of claudins 1, 2, 3, 4, 5 and 7 in various types of tumours. Histopathology **46**(5), 551–60 (2005). doi:10.1111/j.1365-2559.2005.02127.x

[12] Merikallio, H., Pääkkö, P., Harju, T., Soini, Y.: Claudins 10 and 18 are predominantly expressed in lung adenocarcinomas and in tumors of non-smokers. International journal of clinical and experimental pathology **4**(7), 667–73 (2011)

[13] Minoo, P., Su, G., Drum, H., Bringas, P., Kimura, S.: Defects in tracheoesophageal and lung morphogenesis in Nkx2.1(-/-) mouse embryos. Developmental biology **209**(1), 60–71 (1999). doi:10.1006/dbio.1999.9234

[14] Yatabe, Y.M.D., Mitsudomi, T.M.D., Takahashi, T.M.D.: TTF-1 expression in pulmonary adenocarcinomas. The American journal of surgical pathology **26**(6), 767–73 (2002)

[15] Wang, X.-M., Li, J., Yan, M.-X., Liu, L., Jia, D.-S., Geng, Q., Lin, H.-C., He, X.-H., Li, J.-J., Yao, M.: Integrative analyses identify osteopontin, LAMB3 and ITGB1 as critical pro-metastatic genes for lung cancer. PloS one **8**(2), 55714 (2013). doi:10.1371/journal.pone.0055714

[16] Fehrenbach, H., Kasper, M., Tschernig, T., Shearman, M.S., Schuh, D., Müller, M.: Receptor for advanced glycation endproducts (RAGE) exhibits highly differential cellular and subcellular localisation in rat and human lung. Cellular and molecular biology (Noisy-le-Grand, France) **44**(7), 1147–57 (1998)

[17] Jing, R., Cui, M., Wang, J., Wang, H.: Receptor for advanced glycation end products (RAGE) soluble form (sRAGE): a new biomarker for lung cancer. Neoplasma **57**(1), 55–61 (2010)

[18] Corut, A., Senyigit, A., Ugur, S.A., Altin, S., Ozcelik, U., Calisir, H., Yildirim, Z., Gocmen, A., Tolun, A.: Mutations in SLC34A2 cause pulmonary alveolar microlithiasis and are possibly associated with testicular microlithiasis. American journal of human genetics **79**(4), 650–6 (2006). doi:10.1086/508263

[19] Lindskog, C., Fagerberg, L., Hallström, B., Edlund, K., Hellwig, B., Rahnenführer, J., Kampf, C., Uhlén, M., Pontén, F., Micke, P.: The lung-specific proteome defined by integration of transcriptomics and antibody-based profiling. FASEB journal : official publication of the Federation of American Societies for Experimental Biology, 14–254862 (2014). doi:10.1096/fj.14-254862

[20] Geier, C., Perrot, A., Ozcelik, C., Binner, P., Counsell, D., Hoffmann, K., Pilz, B., Martiniak, Y., Gehmlich, K., van der Ven, P.F.M., Fürst, D.O., Vornwald, A., von Hodenberg, E., Nürnberg, P., Scheffold, T., Dietz, R., Osterziel, K.J.: Mutations in the human muscle LIM protein gene in families with hypertrophic cardiomyopathy. Circulation **107**(10), 1390–5 (2003)

[21] Bhavsar, P.K., Brand, N.J., Yacoub, M.H., Barton, P.J.: Isolation and characterization of the human cardiac troponin I gene (TNNI3). Genomics **35**(1), 11–23 (1996). doi:10.1006/geno.1996.0317

[22] Wittenberg, J.B.: Myoglobin function reassessed. Journal of Experimental Biology **206**(12), 2011–2020 (2003). doi:10.1242/jeb.00243

[23] Beqqali, A., Monshouwer-Kloots, J., Monteiro, R., Welling, M., Bakkers, J., Ehler, E., Verkleij, A., Mummery, C., Passier, R.: CHAP is a newly identified Z-disc protein essential for heart and skeletal muscle function. Journal of cell science **123**(Pt 7), 1141–50 (2010). doi:10.1242/jcs.063859

[24] van Spaendonck-Zwarts, K.Y., Posafalvi, A., van den Berg, M.P., Hilfiker-Kleiner, D., Bollen, I.A.E., Sliwa, K., Alders, M., Almomani, R., van Langen, I.M., van der Meer, P., Sinke, R.J., van der Velden, J., Van Veldhuisen, D.J., van Tintelen, J.P., Jongbloed, J.D.H.: Titin gene mutations are common in families with both peripartum cardiomyopathy and dilated cardiomyopathy. European heart journal **35**(32), 2165–73 (2014). doi:10.1093/eurheartj/ehu050

[25] Brenner, M., Johnson, A.B., Boespflug-Tanguy, O., Rodriguez, D., Goldman, J.E., Messing, A.: Mutations in GFAP, encoding glial fibrillary acidic protein, are associated with Alexander disease. Nature genetics **27**(1), 117–20 (2001). doi:10.1038/83679

[26] Gery, S., Yin, D., Xie, D., Black, K.L., Koeffler, H.P.: TMEFF1 and brain tumors. Oncogene **22**(18), 2723–7 (2003). doi:10.1038/sj.onc.1206351

[27] Aizawa, H., Sekine, Y., Takemura, R., Zhang, Z., Nangaku, M., Hirokawa, N.: Kinesin family in murine central nervous system. The Journal of cell biology **119**(5), 1287–96 (1992)

[28] Willemsen, M.H., Ba, W., Wissink-Lindhout, W.M., de Brouwer, A.P.M., Haas, S.A., Bienek, M., Hu, H., Vissers, L.E.L.M., van Bokhoven, H., Kalscheuer, V., Nadif Kasri, N., Kleefstra, T.: Involvement of the kinesin family members KIF4A and KIF5C in intellectual disability and synaptic function. Journal of medical genetics **51**(7), 487–94 (2014). doi:10.1136/jmedgenet-2013-102182

[29] Bahn, S., Mimmack, M., Ryan, M., Caldwell, M.A., Jauniaux, E., Starkey, M., Svendsen, C.N., Emson, P.: Neuronal target genes of the neuron-restrictive silencer factor in neurospheres derived from fetuses with Down's syndrome: a gene expression study. Lancet **359**(9303), 310–5 (2002). doi:10.1016/S0140-6736(02)07497-4

[30] Zhang, X., Zhang, Q.-Y., Liu, D., Su, T., Weng, Y., Ling, G., Chen, Y., Gu, J., Schilling, B., Ding, X.: Expression of cytochrome p450 and other biotransformation genes in fetal and adult human nasal mucosa. Drug metabolism and disposition: the biological fate of chemicals **33**(10), 1423–8 (2005). doi:10.1124/dmd.105.005769

[31] Isaac, J.T.R., Ashby, M.C., McBain, C.J.: The role of the GluR2 subunit in AMPA receptor function and synaptic plasticity. Neuron **54**(6), 859–71 (2007). doi:10.1016/j.neuron.2007.06.001

[32] Hackmann, K., Matko, S., Gerlach, E.-M., von der Hagen, M., Klink, B., Schrock, E., Rump, A., Di Donato, N.: Partial deletion of GLRB and GRIA2 in a patient with intellectual disability. European journal of human genetics : EJHG **21**(1), 112–4 (2013). doi:10.1038/ejhg.2012.97

[33] Kerr, D.I., Ong, J.: GABAB receptors. Pharmacology & therapeutics **67**(2), 187–246 (1995)

[34] Kaupmann, K., Malitschek, B., Schuler, V., Heid, J., Froestl, W., Beck, P., Mosbacher, J., Bischoff, S., Kulik, A., Shigemoto, R., Karschin, A., Bettler, B.: GABA(B)-receptor subtypes assemble into functional heteromeric complexes. Nature **396**(6712), 683–7 (1998). doi:10.1038/25360

[35] Fatemi, S.H., Folsom, T.D., Reutiman, T.J., Thuras, P.D.: Expression of GABA(B) receptors is altered in brains of subjects with autism. Cerebellum (London, England) **8**(1), 64–9 (2009). doi:10.1007/s12311-008-0075-3

[36] Fatemi, S.H., Folsom, T.D., Thuras, P.D.: Deficits in GABA(B) receptor system in schizophrenia and mood disorders: a post-mortem study. Schizophrenia research **128**(1-3), 37–43 (2011). doi:10.1016/j.schres.2010.12.025

[37] Myers, M.W., Lazzarini, R.A., Lee, V.M., Schlaepfer, W.W., Nelson, D.L.: The human mid-size neurofilament subunit: a repeated protein sequence and the relationship of its gene to the intermediate filament gene family. The EMBO journal **6**(6), 1617–26 (1987)

[38] Fernández-Chacón, R., Königstorfer, A., Gerber, S.H., García, J., Matos, M.F., Stevens, C.F., Brose, N., Rizo, J., Rosenmund, C., Südhof, T.C.: Synaptotagmin I functions as a calcium regulator of release probability. Nature **410**(6824), 41–9 (2001). doi:10.1038/35065004

[39] Reddy, P.H., Mani, G., Park, B.S., Jacques, J., Murdoch, G., Whetsell, W., Kaye, J., Manczak, M.: Differential loss of synaptic proteins in Alzheimer's disease: implications for synaptic dysfunction. Journal of Alzheimer's disease : JAD **7**(2), 103–1717380 (2005)

[40] Gosso, M.F., de Geus, E.J.C., van Belzen, M.J., Polderman, T.J.C., Heutink, P., Boomsma, D.I., Posthuma, D.: The SNAP-25 gene is associated with cognitive ability: evidence from a family-based study in two independent Dutch cohorts. Molecular psychiatry **11**(9), 878–86 (2006). doi:10.1038/sj.mp.4001868

[41] Spellmann, I., Müller, N., Musil, R., Zill, P., Douhet, A., Dehning, S., Cerovecki, A., Bondy, B., Möller, H.-J., Riedel, M.: Associations of SNAP-25 polymorphisms with cognitive dysfunctions in Caucasian patients with schizophrenia during a brief trail of treatment with atypical antipsychotics. European archives of psychiatry and clinical neuroscience **258**(6), 335–44 (2008). doi:10.1007/s00406-007-0800-9

[42] Nishihara, S., Iwasaki, H., Nakajima, K., Togayachi, A., Ikehara, Y., Kudo, T., Kushi, Y., Furuya, A., Shitara, K., Narimatsu, H.: Alpha1,3-fucosyltransferase IX (Fut9) determines Lewis X expression in brain. Glycobiology **13**(6), 445–55 (2003). doi:10.1093/glycob/cwg048

[43] Serafini-Cessi, F., Malagolini, N., Hoops, T.C., Rindler, M.J.: Biosynthesis and oligosaccharide processing of human Tamm-Horsfall glycoprotein permanently expressed in HeLa cells. Biochemical and biophysical research communications **194**(2), 784–90 (1993)

[44] Béguin, P., Wang, X., Firsov, D., Puoti, A., Claeys, D., Horisberger, J.D., Geering, K.: The gamma subunit is a specific component of the Na,K-ATPase and modulates its transport function. The EMBO journal **16**(14), 4250–60 (1997)

[45] Arystarkhova, E., Wetzel, R.K., Asinovski, N.K., Sweadner, K.J.: The gamma subunit modulates Na(+) and K(+) affinity of the renal Na,K-ATPase. The Journal of biological chemistry **274**(47), 33183–5 (1999)

[46] Thomson, R.B., Igarashi, P., Biemesderfer, D., Kim, R., Abu-Alfa, A., Soleimani, M., Aronson, P.S.: Isolation and cDNA cloning of Ksp-cadherin, a novel kidney-specific member of the cadherin multigene family. The Journal of biological chemistry **270**(29), 17594–601 (1995)

[47] Thomson, R.B., Aronson, P.S.: Immunolocalization of Ksp-cadherin in the adult and developing rabbit kidney. The American journal of physiology **277**(1 Pt 2), 146–56 (1999)

[48] Shen, S.S., Krishna, B., Chirala, R., Amato, R.J., Truong, L.D.: Kidney-specific cadherin, a specific marker for the distal portion of the nephron and related renal neoplasms. Modern pathology : an official journal of the United States and Canadian Academy of Pathology, Inc **18**(7), 933–40 (2005). doi:10.1038/modpathol.3800373

[49] Kocher, O., Cheresh, P., Brown, L., Lee, S.: Identification of a novel gene, selectively up-regulated in human carcinomas, using the differential display technique. Clin. Cancer Res. **1**(10), 1209–1215 (1995)

[50] Kocher, O., Cheresh, P., Lee, S.W.: Identification and partial characterization of a novel membrane-associated protein (MAP17) up-regulated in human carcinomas and modulating cell replication and tumor growth. The American journal of pathology **149**(2), 493–500 (1996)

[51] Schwelberger, H.G., Bodner, E.: Identity of the diamine oxidase proteins in porcine kidney and intestine. Inflammation research : official journal of the European Histamine Research Society ... [et al.] **47 Suppl 1**, 58–9 (1998)

[52] Morel, F., Surla, A., Vignais, P.V.: Purification of human placenta diamine oxidase. Biochemical and biophysical research communications **187**(1), 178–86 (1992)

[53] Biegaski, T., Kusche, J., Lorenz, W., Hesterberg, R., Stahlknecht, C.D., Feussner, K.D.: Distribution and properties of human intestinal diamine oxidase and its relevance for the histamine catabolism. Biochimica et biophysica acta **756**(2), 196–203 (1983)

[54] Yu, A.S.L., Enck, A.H., Lencer, W.I., Schneeberger, E.E.: Claudin-8 expression in Madin-Darby canine kidney cells augments the paracellular barrier to cation permeation. The Journal of biological chemistry **278**(19), 17350–9 (2003). doi:10.1074/jbc.M213286200

[55] Cha, S.H., Sekine, T., Fukushima, J.I., Kanai, Y., Kobayashi, Y., Goya, T., Endou, H.: Identification and characterization of human organic anion transporter 3 expressing predominantly in the kidney. Molecular pharmacology **59**(5), 1277–86 (2001)

[56] Ferrari, P.: The role of 11β-hydroxysteroid dehydrogenase type 2 in human hypertension. Biochimica et biophysica acta **1802**(12), 1178–87 (2010). doi:10.1016/j.bbadis.2009.10.017

Predicted marker genes for liver

Gene Symbol	Gene Full Name
AKR1D1	aldo-keto reductase family 1, member D1
FGG	fibrinogen gamma chain
APOA2	apolipoprotein A-II
CYP2C8	cytochrome P450, family 2, subfamily C, polypeptide 8
GC	group-specific component (vitamin D binding protein)
CPS1	carbamoyl-phosphate synthase 1, mitochondrial
CYP2E1	cytochrome P450, family 2, subfamily E, polypeptide 1
APOC3	apolipoprotein C-III
SERPINC1	serpin peptidase inhibitor, clade C (antithrombin), member 1
AHSG	alpha-2-HS-glycoprotein
AMBP	alpha-1-microglobulin/bikunin precursor

Predicted marker genes for lung

Gene Symbol	Gene Full Name
CLDN18	claudin 18
NKX2-1	NK2 homeobox 1
SCGB1A1	secretoglobin, family 1A, member 1
SFTPB	surfactant protein B
CYP4B1	cytochrome P450, family 4, subfamily B, polypeptide 1
CD52	CD52 molecule
LAMP3	lysosomal-associated membrane protein 3
AGER	advanced glycosylation end product-specific receptor
LYZ	lysozyme
SFTPD	surfactant protein D
SFTPC	surfactant protein C
SLC34A2	solute carrier family 34 (type II sodium/phosphate contransporter), member 2

Predicted marker genes for heart

Gene Symbol	Gene Full Name
MYOZ2	myozenin 2
TNNI3	troponin I type 3 (cardiac)
SYNPO2L	synaptopodin 2-like
MYH6	myosin, heavy chain 6, cardiac muscle, alpha
CSRP3	cysteine and glycine-rich protein 3 (cardiac LIM protein)
CKM	creatine kinase, muscle
PLN	phospholamban
MB	myoglobin
TTN	titin
MYL7	myosin, light chain 7, regulatory
MYH7	myosin, heavy chain 7, cardiac muscle, beta
TPM1	tropomyosin 1 (alpha)

Predicted marker genes for brain

Gene Symbol	Gene Full Name
GAP43	growth associated protein 43
GFAP	glial fibrillary acidic protein
TMEFF1	transmembrane protein with EGF-like and two follistatin-like domains 1
FUT9	fucosyltransferase 9 (alpha (1,3) fucosyltransferase)
SYT1	synaptotagmin I
SNAP25	synaptosomal-associated protein, 25kDa
MBP	myelin basic protein
GRIA2	glutamate receptor, ionotropic, AMPA 2
KIF5C	kinesin family member 5C
STMN2	stathmin 2
NEFM	neurofilament, medium polypeptide
GABBR2	gamma-aminobutyric acid (GABA) B receptor, 2

Predicted marker genes for kidney

Gene Symbol	Gene Full Name
SLC12A1	solute carrier family 12 (sodium/potassium/chloride transporter), member 1
SLC3A1	solute carrier family 3 (amino acid transporter heavy chain), member 1
UMOD	uromodulin
AOC1	amine oxidase, copper containing 1
CD24	CD24 molecule
HSD11B2	hydroxysteroid (11-beta) dehydrogenase 2
CA12	carbonic anhydrase XII
PDZK1IP1	PDZK1 interacting protein 1
FXYD2	FXYD domain containing ion transport regulator 2
CDH16	cadherin 16, KSP-cadherin
SLC22A8	solute carrier family 22 (organic anion transporter), member 8
CLDN8	claudin 8
