# Supplementary material for: MGFM: a novel tool for detection of tissue and cell specific marker genes from microarray gene expression data
Source: BMC Genomics. 2015 Aug 28;16(1):645. doi: 10.1186/s12864-015-1785-9 (PMC4552366; doi:10.1186/s12864-015-1785-9)
Supplement: Additional file 5 — Primer sequences. This file includes the list of all primer sequences used by PCR. (55.7KB PDF) [file 12864_2015_1785_MOESM5_ESM.pdf]

Primer Sequences for PCR amplification

Primers used for liver

Gene name	forward primer (5'-3')	Reverse primer (3'-5')	Product size
AKR1D1	TCTCAGTGCTGCAAGTCACC	CTCCCCAACTTCGTGTTCAT	193
FGG	GAATTTTGGCTGGGAAATGA	ATCATCGCCAAAATCAAAGC	222
APOA2	GAGCTTTGGTTCGGAGACAG	TGTGTTCCAAGTTCCACGAA	235
CYP2C8	GCAGGAAAAGGACAACCAAA	GTGTAAGGCATGTGGCTCCT	228
GC	GAAACACCAGCCACAGGAAT	GGTACAGCAGGACCCTACCA	196
CPS1	ATTCCTTGGTGTGGCTGAAC	ATGGAAGAGAGGCTGGGATT	150
CYP2E1	ACCCGAGACACCATTTTCAG	TCCAGCACACACTCGTTTTC	201
APOC3	CTCCCTTCTCAGCTTCATGC	GTCTGACCTCAGGGTCCAAA	200
SERPINC1	TTTACTTCAAGGGCCTGTGG	CTTTGAAGGGCAACTCAAGC	171
AHSG	CAGAACAACGGCTCCAATTT	CTGTGTTTGGAACACCATGC	240
AMBP	AGTGGTACAACCTGGCCATC	AAGCTCCAGACGTCTCCTCA	165

Primers used for lung

Gene name	forward primer (5'-3')	Reverse primer (3'-5')	Product size
CLDN18	GGTATCCATCTTTGCCCTGA	GGTCTGAACAGTCTGCACCA	217
NKX2-1	ACAAGAAAGTGGGCATGGAG	GTTCCTCATGGTGTCCTGGT	248
SCGB1A1	GTCACACTGGCTCTCTGCTG	TGATGCTTTCTCTGGGCTTT	205
SFTPB	CAAACGGCATCTGTATGCAC	CGGAGAGATCCTGTGTGTGA	195
CYP4B1	CCTGGACAAAGTGGTGTCCT	CCAATCCACTGGAGGAAGAA	171
CD52	GCGCTTCCTCTTCCTCCTAC	GAGGTGGATTATGGCATTGG	166
LAMP3	ACTTCAACATCGACCCCAAC	CACTCACGCACTTGAAGGAA	246
AGER	GCTGTCAGCATCAGCATCAT	ATTCAGTTCTGCACGCTCCT	225
LYZ	GCCAAATGGGAGAGTGGTTA	ATCACGGACAACCCTCTTTG	213
SFTPD	AAGTGGGCTTCCAGATGTTG	CTGTGCCTCCGTAAATGGTT	181
SFTPC	ACACTGCCACCTTCTCCATC	CTGGCCCAGCTTAGACGTAG	221
SLC34A2	TCGCCACTGTCATCAAGAAG	TGGATAAGCCCTCTCAATGG	185

Primers used for heart

Gene name	forward primer (5'-3')	Reverse primer (3'-5')	Product size
MYOZ2	CCGGAGCTTTTAGAGGCTTT	CCAGAAAGGGGATTGACAAA	206
TNNI3	TGCAGATTGCAAAGCAAGAG	CAGATCTGCAATCTCCGTGA	224
SYNPO2L	CGACCTGGTCCTCATCTCAT	GCCGCCGTTTCTTAAACATA	232
MYH6	CTTCAACCACCACATGTTCG	GGCTTCTGGAAATTGTTGGA	234
CSRP3	CCTTGGCACAAGACCTGTTT	TTGTGTAAGGCCTCCAAACC	150
CKM	TGAAAACCTCAAGGGTGGAG	TCCTTCTCCGTCATGCTCTT	213
PLN	GAGAAAGTCCAATACCTCACTCG	GAGAAGCATCACGATGATACAGA	153
MB	GAGATGAAGGCGTCTGAGGA	TCTGCAGAACCTGGATGATG	190
TTN	AGGCTGGAAACGGTGTAATG	TAGGGCATCCTGCTCTCACT	233
MYL7	GTCTTCCTCACGCTCTTTGG	CCACCTCAGCTGGAGAGAAC	166
MYH7	TGTGTCACCGTCAACCCTTA	TGGCTGCAATAACAGCAAAG	238
TPM1	AGTCGAGCCCAAAAAGATGA	TTCTTCCAGCTGTCGGACTT	192

Primers used for brain

Gene name	forward primer (5'-3')	Reverse primer (3'-5')	Product size
GAP43	GGGAGGCTTGAGGAAAAATC	TCAGCAGCTTGGACATCATC	240
GFAP	AGGAAGATTGAGTCGCTGGA	ATACTGCGTGCGGATCTCTT	165
TMEFF1	CCCTGTTTTGTTCGAGAAGC	CACATTTTCCATGGATGCAG	229
FUT9	CAAAAGAGTGGCATTGAGCA	AATGCTTGCCCGTAGGTATG	236
SYT1	GTGAGCGAGAGTCACCATGA	ACGGTGGCAATGGAATTTTA	172
SNAP25	ATGCCCGAGAAAATGAAATG	AGCATCTTTGTTGCACGTTG	190
MBP	GGGAGGACAACACCTTCAAA	ACTTGCTGTGGCCAGGTACT	155
GRIA2	GTGGCTAGAGTGCGGAAGTC	ACGCCTTGCTCACTGAGTTT	206
KIF5C	TGCAGGATGCTGAAGAAATG	GCTTCTCCTGTTCCAGTTGC	168
STMN2	AGCTGTCCATGCTGTCACTG	CAGCCTCCAGTTTCTTCTGG	231
NEFM	TCAACGTCAAGATGGCTCTG	CTTCCACCTTGGGTTTCTGA	165
GABBR2	CGCCTGTTCTAGCCGATAAG	GGTACAGGGATCGTTGGAGA	236

Primers used for kidney

Gene name	forward primer (5'-3')	Reverse primer (3'-5')	Product size
SLC12A1	TTTGGAGCTGTTTTGTGCTG	ATGGGTCCCCCTGTTAAGAC	245
SLC3A1	GGGAACAGCGTGTATGAGGT	GGAGTTCCAGGGAGTGTGAA	167
UMOD	AAGAGTCTGGGCTTCGACAA	GCTGTAAGTGGCATGGGTTT	162
AOC1	AGGCATGCAGACCAAGTACC	GGCATTTCAAAGAGGCAGAG	171
CD24	ACCCACGCAGATTTATTCCA	ACCACGAAGAGACTGGCTGT	153
HSD11B2	GACCTGACCAAACCAGGAGA	GCCAAAGAAATTCACCTCCA	174
CA12	AGTACAAAGGCCAGGAAGCA	GCCGGAAGTTGTTGATCATT	246
PDZK1IP1	TTGCAGTCAACCACTTCTGG	GCATTCTCATGCTCACTGGA	151
FXYD2	CGTGGACCCGTTCTACTATGA	GGCTCATCTTCATTGATTTGC	153
CDH16	GGGAAACCTCTACGTGACCA	AGCTCAGGGATGCTGACTGT	189
SLC22A8	CTGAGCACCGTCATCTTGAA	GACCAACCAGCGTATGGACT	219
CLDN8	GGGGACAATGAGAAGGTGAA	GAGCTCCTCCAACAATCAGC	217
